# Multi-omics analyses of CD276 in pan-cancer reveals its clinical prognostic value in glioblastoma and other major cancer types

**DOI:** 10.1186/s12885-023-10575-1

**Published:** 2023-01-30

**Authors:** Lirui Dai, Xuyang Guo, Zhe Xing, Yiran Tao, Wulong Liang, Zimin Shi, Weihua Hu, Shaolong Zhou, Xinjun Wang

**Affiliations:** 1 Department of Neurosurgery, The Fifth Affiliated Hospital of Zhengzhou University, Zhengzhou University, Zhengzhou, 450052 China; 2 Institute of Neuroscience, Zhengzhou University, Zhengzhou, 450052 China; 3 Henan International Joint Laboratory of Glioma Metabolism and Microenvironment Research, Zhengzhou, Henan China

**Keywords:** Pan-cancer, Single-cell analysis, Bioinformatics, Immune microenvironment, *CD276*, Immune checkpoints, Glioblastoma

## Abstract

**Background:**

*CD276* (also known as *B7-H3*) is one of the most important immune checkpoints of the *CD28* and B7 superfamily, and its abnormal expression is closely associated with various types of cancer. It has been shown that *CD276* is able to inhibit the function of T cells, and that this gene may potentially be a promising immunotherapy target for different types of cancer.

**Methods:**

Since few systematic studies have been published on the role of *CD276* in cancer to date, the present study has employed single-cell sequencing and bioinformatics methods to analyze the expression patterns, clinical significance, prognostic value, epigenetic alterations, DNA methylation level, tumor immune cell infiltration and immune functions of *CD276* in different types of cancer. In order to analyze the potential underlying mechanism of *CD276* in glioblastoma (GBM) to assess its prognostic value, the LinkedOmics database was used to explore the biological function and co-expression pattern of *CD276* in GBM, and Gene Ontology (GO) and Kyoto Encyclopedia of Genes and Genomes (KEGG) enrichment analyses were performed. In addition, a simple validation of the above analyses was performed using reverse transcription-quantitative (RT-q)PCR assay.

**Results:**

The results revealed that *CD276* was highly expressed, and was often associated with poorer survival and prognosis, in the majority of different types of cancer. In addition, *CD276* expression was found to be closely associated with T cell infiltration, immune checkpoint genes and immunoregulatory interactions between lymphoid and a non-lymphoid cell. It was also shown that the *CD276* expression network exerts a wide influence on the immune activation of GBM. The expression of *CD276* was found to be positively correlated with neutrophil-mediated immunity, although it was negatively correlated with the level of neurotransmitters, neurotransmitter transport and the regulation of neuropeptide signaling pathways in GBM. It is noteworthy that *CD276* expression was found to be significantly higher in GBM compared with normal controls according to the RT-qPCR analysis, and the co-expression network, biological function and chemotherapeutic drug sensitivity of *CD276* in GBM were further explored. In conclusion, the findings of the present study have revealed that *CD276* is strongly expressed and associated with poor prognosis in most types of cancer, including GBM, and its expression is strongly associated with T-cell infiltration, immune checkpoint genes, and immunomodulatory interactions between lymphocytes and non-lymphoid cells.

**Conclusions:**

Taken together, based on our systematic analysis, our findings have revealed important roles for *CD276* in different types of cancers, especially GBM, and *CD276* may potentially serve as a biomarker for cancer.

**Supplementary Information:**

The online version contains supplementary material available at 10.1186/s12885-023-10575-1.

## Introduction

Cancer is currently the leading cause of human death globally, and increased rates of morbidity and mortality have been identified in recent years. The latest data released by the International Agency for Research on Cancer in 2020 showed that lung cancer remains the most common cause of cancer-associated death, followed by colorectal cancer and prostate cancer, with breast cancer being the most common cause of death among women [1]. There is therefore an urgent need to identify effective preventative measures, diagnostic markers and therapeutic methods to control the spread of cancer. Interfering with the *PD-1* (programmed cell death protein 1) and *CTLA-4* (cytotoxic T-lymphocyte-associated antigen 4) immune checkpoints has been routinely used in tumor immunotherapy, although, as a recently discovered immune checkpoint in tumor immunotherapy, an increasing number of studies have focused on the role of *CD276 (B7-H3)* [2].

Cancerous cells are in an unregulated state, and cancer progression requires inhibition of damage surveillance mechanisms and the positive regulation of genetic and epigenetic instability factors in order to achieve uncontrolled cell proliferation and aggressiveness [3]. There is some evidence that members of the B7 family of immune checkpoint proteins can stimulate and enhance the effects of T-cells, while inhibiting and attenuating negative signals from T-cell responses in human cancers [4]. As immune checkpoint molecules, B7 family proteins can inhibit T-cell-mediated immune surveillance via binding to inhibitory receptors; they also participate in the immune evasion of tumor cells, thereby promoting tumor progression [5]. At present, blocking B7 ligand inhibitory interactions to improve T-cell infiltration is routinely used as a strategy to guide tumor immunotherapy, especially *PD-1* and *CTLA-4* blocking antibodies in renal cancer and melanoma [6]. As a member of the *CD28* and B7 families, *CD276* inhibits T-cell function, and is highly expressed in numerous types of tumor, so it is often associated with poor prognosis and shorter survival rates in cancer patients [7]. At present, the molecular mechanism underlying *CD276* expression regulation is becoming increasingly well understood, as it has been shown to promote tumor activity and to enhance cell proliferation, migration, invasion, angiogenesis and chemotherapy resistance both in vivo and in vitro [8]. For example, Wang et al. [9] found that *CD276* is able to promote colorectal cancer angiogenesis via activating the *NF-κB* pathway to induce the expression of vascular endothelial growth factor A (*VEGFA*). A previously published study also revealed that *CD276* may regulate glucose metabolism and chemotherapy resistance by controlling the expression of hexokinase 2 (*HK2*) in colorectal cancer cells [10]. In addition, Yuan et al. [11] found that the expression level of *CD276* in a prostate cancer group was significantly higher compared with that in the associated benign prostatic hyperplasia group, and knockdown of *CD276* led to a marked reduction in the migratory and invasive capabilities of prostate cancer cells, thereby demonstrating that *CD276* has an important role in the progression of prostate cancer. In addition, *CD276* was also shown to affect the glycolytic ability of cancer cells through regulating the activity of metabolic enzymes [12]. As a negative regulator of T-cell function, *CD276* acts preferentially on type 1 T helper cells [13]. Co-culture of myeloid dendritic cells and regulatory T-cells led to an upregulation of the expression of *CD276*, thereby regulating the maturation of dendritic cells [14]. In addition, in another study, microRNAs (miRNAs) were also shown to regulate *CD276* expression via interacting with the 3’-UTR (3’-untranslated region) of the *CD276* mRNA [15].

Many different researchers have also shown that *CD276* has a crucial role in the progression of most types of cancer. A recently published study showed that *CD276* is highly expressed by cancer stem cells (CSCs) in mouse and human head and neck squamous cell carcinoma, thereby facilitating immune escape and promoting tumor growth and lymph node metastasis [16]. The pro-oncogenic kinase *PBK* was also shown to promote *MSL* phosphorylation on *CD276*, and to activate *CD276* transcription in nasopharyngeal carcinoma. In addition, as an immune checkpoint molecule, the increased expression of *CD276* was shown to be associated with the level of immune infiltration in nasopharyngeal carcinoma [17]. Furthermore, *ILT4* (immunoglobulin-like transcript 4) has been shown to increase *CD276* expression in non-small cell lung cancer via the *PI3K/AKT/mTOR* signaling pathway [18]. In conclusion, a range of studies have collectively shown that the expression of *CD276* in different types of cancer can be regulated by numerous interconnected factors, although the function and potential molecular mechanism of *CD276* in tumor progression requires further study.

Although an increasing number of studies have revealed that *CD276* is able to influence cancer progression via various molecular mechanisms, to date, no comprehensive pan-cancer analysis of *CD276* has been carried out, and few single-cell analyses of *CD276* in different types of cancer have been performed [16]. Hence, in the present study, we have systematically analyzed the expression patterns, single-cell levels in human tissue, expression levels in cell subpopulations and gene expression of *CD276* in different cell types of different brain tissues; moreover, the expression profile of *CD276* in 33 types of cancer, the prognostic value of cancer patients, epigenetic alterations, DNA methylation levels, and the association between *CD276* and immune-activating genes, immunosuppressive genes, chemokines, chemokine receptors and immune checkpoints have been investigated.

Glioblastoma (GBM), the most aggressive and common brain tumor in adults, is characterized by an extremely poor prognosis, significantly high tumor heterogeneity, and limited treatment options. An increasing amount of high-throughput data are shedding light on the genomic or epigenetic profile of GBM, and are guiding tumor exploration and treatment according to the cellular components and key molecules that drive the most aggressive behavior [19]. In the present study, we have mainly analyzed the co-expression network, biological function and susceptibility to chemotherapeutic drugs of *CD276* in GBM, and have also explored the potential correlation between *CD276* expression and immune function. The results obtained have provided both novel insights into the functional role of *CD276* in various types of tumor, especially in the case of GBM, and new knowledge that should be beneficial for the optimization of cancer immunotherapy.

## Materials and methods

### Single-cell analysis

Human Cell Landscape (http://bis.zju.edu.cn/HCL/) [20] and PanglaoDB (https://panglaodb.se/index.html) [21] databases were used to analyze the cell types of human embryos and adults, and the expression levels of *CD276* in various types of tissue. The expression levels of *CD276* in adult and fetal brain tissues, and different cell types therein, were analyzed. Subsequently, cell aggregation and *CD276* expression in brain cell subsets were further analyzed using the Tabula Muris database (https://tabula-muris.ds.czbiohub.org/) [22]. In addition, CancerSEA (http://biocc.hrbmu.edu.cn/CancerSEA/home.jsp) [23] and TIGER (http://tiger.canceromics.org/) [24] databases were used to explore the expression levels and distribution of *CD276* expression in pan-cancer, including GBM, so as to comprehensively analyze the functions of cancer cells at the single-cell resolution.

### *CD276* mRNA expression in pan-cancer

The Cancer Genome Atlas (TCGA; https://www.cancer.gov/) [25] database was used to download the data of 33 types of cancer, and *CD276* expression was studied in the 33 different cancer types. In addition, the Genotype-Tissue Expression (GTEx, https://commonfund.nih.gov/GTEx/) [26] dataset was searched to obtain data of normal tissues. Furthermore, *CD276* expression was evaluated in various cancer cell lines using the cBioPortal database (https://www.cbioportal.org/) [27].

### *CD276* protein expression in pan-cancer

The Human Protein Atlas (HPA: https://www.proteinatlas.org/) [28] dataset is an open access resource for human proteins containing 12 different sections. The pathology section enables an exploration of the expression profiles of human cancers to be made. This section contains pathology information based on mRNA and protein expression data from 17 different forms of human cancer, together with millions of immunohistochemically stained tissue section images generated in-house and Kaplan-Meier plots showing the correlation between mRNA expression of each human protein gene and cancer patient survival. This section of the HPA dataset was explored, and the protein expression levels of *CD276* in cancerous and normal tissues were evaluated. The subcellular section of the HPA dataset contains a subcellular map of the human proteome, and provides high-resolution information on the expression and spatiotemporal distribution of proteins encoded by 13,105 genes. For each gene, the subcellular distribution of the protein has been investigated by immunofluorescence and confocal microscopy experiments in up to three different cell lines, selected from a subset of 37 of the cell lines found in the cell line section. Given this background,we explored the subcellular localization of *CD276* and its localization in different cancer cell lines. Furthermore, the GPS-Prot (http://gpsprot.org/) [29], GeneMANIA (http://genemania.org/) [30] and STRING (https://string-db.org/) [31] databases were utilized to establish the protein–protein interaction network (PPI) of *CD276*. Gene Ontology (GO) and Kyoto Encyclopedia of Genes and Genomes (KEGG) enrichment analyses of *CD276* co-expressed genes were performed using the Database for Annotation, Visualization and Integrated Discovery (DAVID) (https://david.ncifcrf.gov/) [32].

### *CD276* expression and clinicopathological characteristics in pan-cancer

The University of ALabama at Birmingham CANcer data analysis portal (UALCAN; http://ualcan.path.uab.edu/) [33] is a comprehensive, user-friendly and interactive web resource for analyzing cancer-omics data. UALCAN has been designed to: a) provide graphs and plots depicting the expression profiles and patient survival information for protein-coding genes; b) evaluate epigenetic regulation of gene expression by promoter methylation; c) provide additional information on the selected genes/targets by linking to the HPRD, GeneCards, PubMed, TargetScan, HPA, DRUGBANK, Open Targets and GTEx databases, enabling researchers to gather valuable information about the genes of interest; and d) provide clinical proteomic consortium data analysis, including the analysis of total/phospho-proteins. In this study, the UALCAN database was used to explore both *CD276* expression in different stages of cancer and the DNA methylation status of *CD276* in different types of cancer.

### Genetic alterations of *CD276* in pan-cancer and comprehensively analysis in GBM

The cBioPortal database was employed to explore the genetic alterations, gene alteration frequencies and copy-number alterations of *CD276* in different types of cancer. The DiseaseMeth version 2.0 (http://bio-bigdata.hrbmu.edu.cn/diseasemeth/) [34] and UALCAN databases were employed to explore differences in the methylation status of *CD276* comparing between GBM and normal tissues [35]. In addition, the MEXPRESS (https://mexpress.be) [36] database was used to explore the association between *CD276* expression and methylation. Finally, the MethSurv (https://biit.cs.ut.ee/methsurv/) [37] database was used for the multivariate survival analysis to assess the CpG island composition and expression of previously undescribed genes, and their association with the survival outcome.

### The prognostic value of *CD276* in pan-cancer

The survival data pertaining to different types of cancer were acquired from TCGA cohort, and the overall survival (OS), disease-specific survival (DSS) and progression-free interval (PFI) rates of patients were analyzed according to the Kaplan-Meier method of analysis. The analysis results were supplemented using the Kaplan-Meier Plotter (http://www.kmplot.com/analysis/) [38] database. In addition, univariate Cox regression analysis was used to explore the value of *CD276* in predicting the OS, DSS and PFI rates of various types of cancer.

### Gene Set Enrichment Analysis (GSEA)

TCGA cohort was used to identify *CD276*-associated genes and to perform correlation analysis. Genes associated with *CD276* were selected for GSEA using the Pearson correlation coefficient calculation (*P*<0.05). All analyses were carried out utilizing DAVID Bioinformatics Resources 6.8 (https://david.ncifcrf.gov/) [39].

### Correlation between *CD276* and tumor immune cell infiltration in pan-cancer

The immune cell infiltration data of different types of cancer in TCGA were acquired from the TIMER2.0 (http://timer.cistrome.org/) [40], CIBERSOFT and TISDB (http://cis.hku.hk/TISIDB/index.php) [41] databases. TIMER2.0 is a comprehensive resource for systematical analysis of immune infiltrates across diverse cancer types. Differences in *CD276* expression among specific tumor subclasses and para-carcinoma tissues in TCGA database were analyzed using the TIMER2.0 database. The “Gene_DE module” enabled an exploration of the differential expression between tumor and adjacent normal tissues for *CD276* across TCGA cancers to be made. The distributions of gene expression levels are shown using box plots. Statistical significances computed using the Wilcoxon test are shown annotated by the number of stars (*, *P*-value <0.05; **, *P*-value <0.01; and ***, *P*-value <0.001). Subsequently, “Immune module” enabled an exploration of the immune infiltration estimations for *CD276* expression to be made using TIMER algorithms. Six immune cell types were analyzed using TIMER algorithms, including lymphocytes, macrophages, NK (natural killer) cells and neutrophils. Each cancer in the heatmap was separated into two groups, namely the low-expression and high-expression *CD276* groups, which were analyzed for immune cell infiltration.

### Relationship Between *CD276* and Immunoregulation-associated genes

The GDC (https://gdc.cancer.gov/) [42] and TISDB databases were used to obtain TCGA data on 33 different types of human cancer. The potential of *CD276* for immunotherapy of human cancer was explored by analyzing the correlation between *CD276* and immune-activating genes, immunosuppressive state genes, chemokine genes and chemokine receptor genes. The horizontal and vertical axes represent the type of cancer and the corresponding gene, respectively, whereas the colors represent the degree of correlation between *CD276* and the associated gene.

### Linked omics database analysis

The LinkedOmics database (http://www.linkedomics.org/login.php) [43] contains multi-omics data and clinical data for all 32 TCGA cancer types, as well as data for a total of 11,158 patients from the TCGA project. It is also a multi-omics database that integrates proteomics data from mass spectrometry for selected TCGA tumor samples. The differentially expressed genes (DEGs) associated with *CD276* were screened from TCGA-GBM cohort using the LinkFinder module, and Pearson’s correlation coefficient was used to analyze the results. In addition, the biological function of *CD276* in GBM was analyzed using GSEA.

### Correlation analysis of gene expression with therapeutic effects

The ROC plotter (http://rocplot.org/) and PubChem (https://pubchem.ncbi.nlm.nih.gov/) databases were used to explore the association between *CD276* expression and therapeutic responses in patients with GBM [44, 45].

### Reverse transcription-quantitative PCR (RT-qPCR) analysis

Eighteen patients treated at the Fifth Affiliated Hospital of Zhengzhou University between 2016 to 2020 were randomly selected to analyze their *CD276* mRNA expression patterns. All patients were over 60 years of age, and those with normal brain tissue were epileptics. Total RNA from patients’ tissues (nine tissues from patients with GBM and nine normal tissues) was extracted using TRIzol™ (Takara Biotechnology Co., Ltd.), and various other reagents, including chloroform, isopropyl alcohol and 75% alcohol. A PrimeScript™ RT reagent kit with gDNA Eraser and TB Green Premix Ex Taq™ II (Takara Biotechnology Co., Ltd.) was employed for reverse-transcription of the RNA into cDNA. The primer sequences were as follows: *CD276*, forward primer, 5’-AGCTGTGAGGAGGAGAATGC-3’, reverse primer, 5’-TGCTGTCAGAGTGTTTCAGAGG-3’; proliferating cell nuclear antigen (*PCNA*), forward primer, 5’-TGGAGAACTTGGAAATGGAAA-3’, reverse primer, 5’-GAACTGGTTCATTCATCTCTATGG-3’; and GAPDH, forward primer, 5’-CAGGAGGCATTGCTGATGAT-3’, reverse primer, 5’- GAAGGCTGGGGCTCATTT -3’.

### Statistical analyses

R 3.6.2 for Windows (R Project) was employed to analyze the data, and Student’s t-test was applied to explore the differences between groups. *P*<0.05 was considered to indicate a statistically significant value.

## Results

### Single cell sequencing of *CD276* expression in tumor and normal tissues

**Fig. 1:**
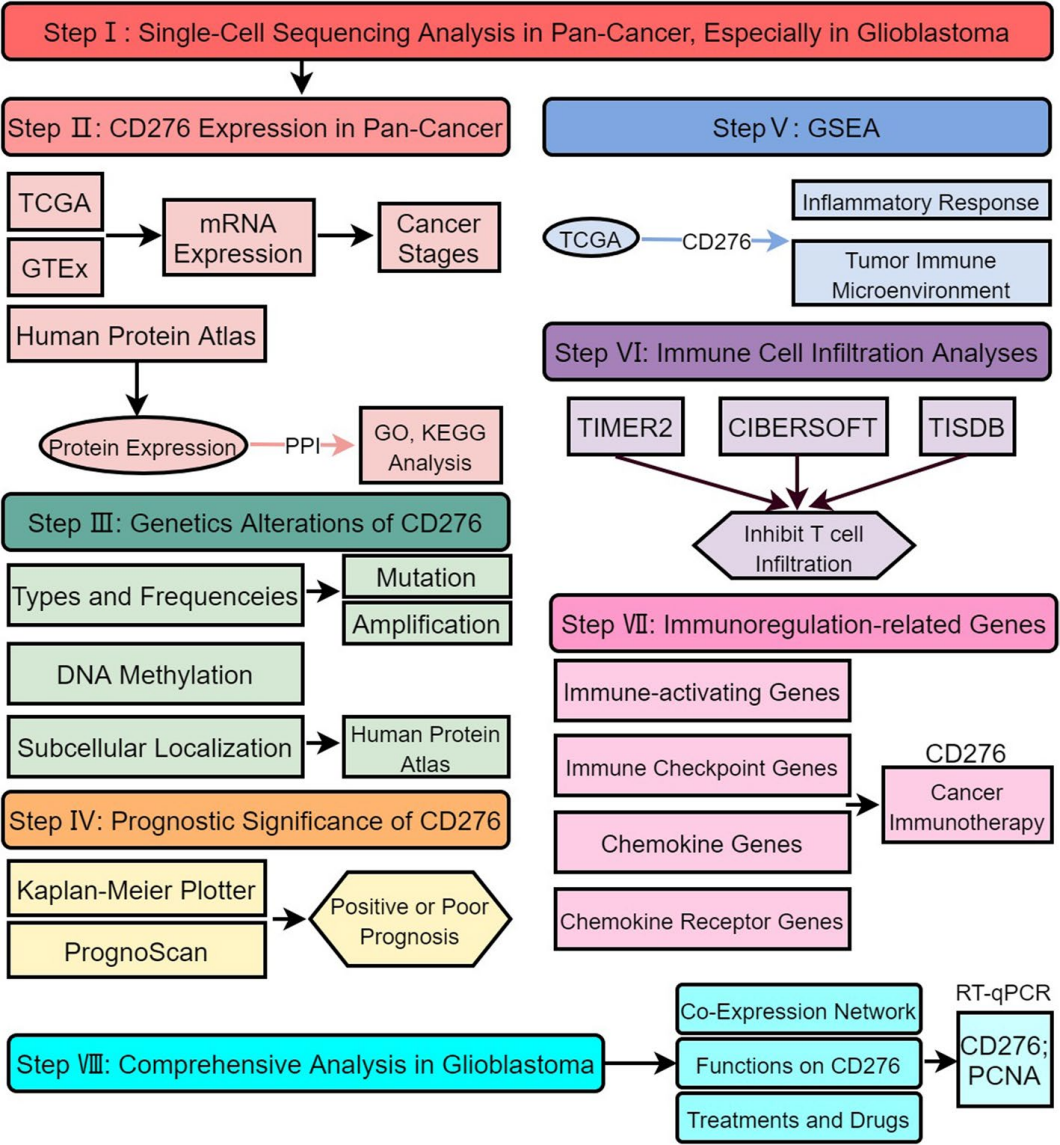
The flowchart of this study

**Fig. 2:**
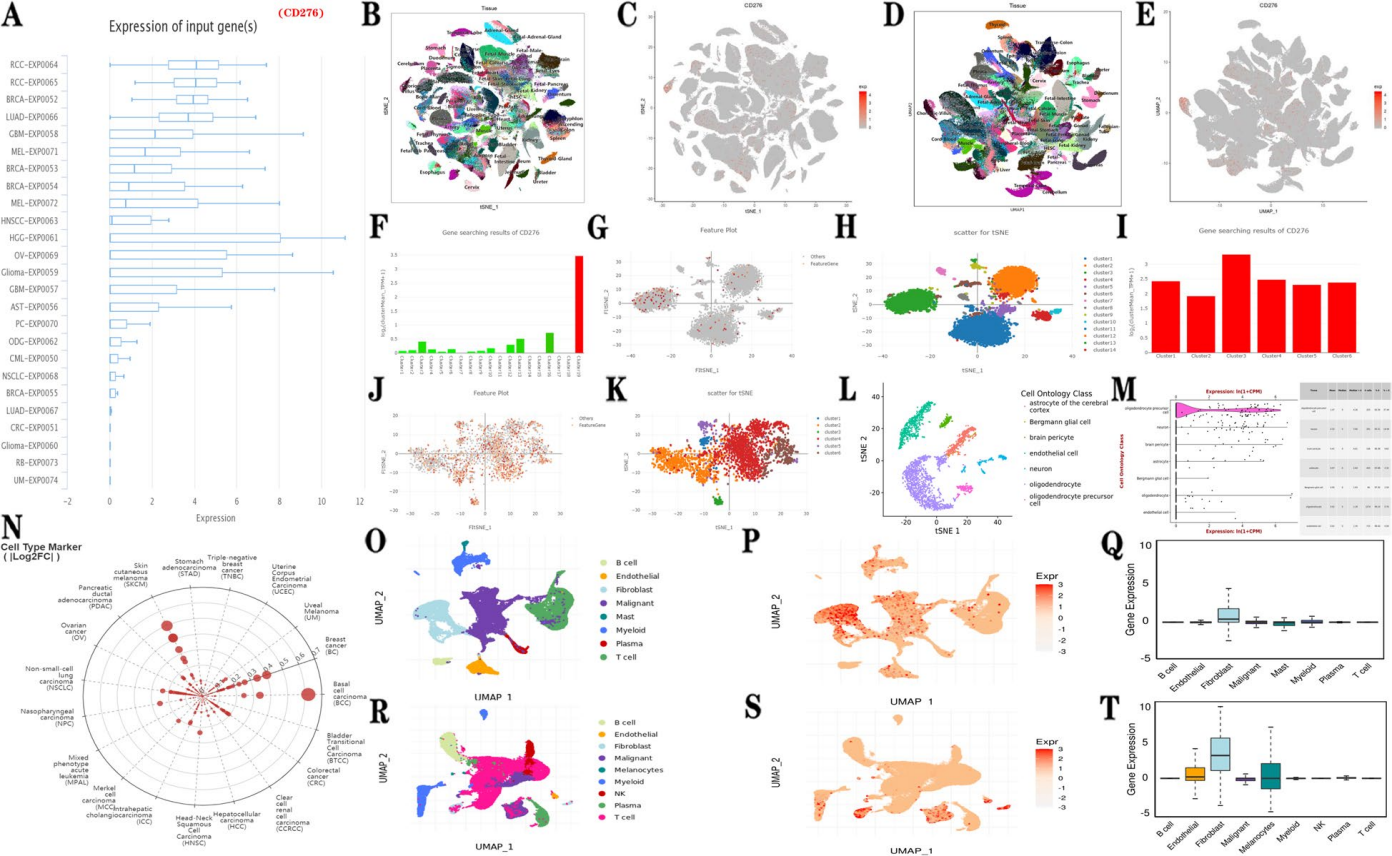
Single-cell analysis. **A** The expression pattern of *CD276* in pan-cancer is shown. **B-E** Single-cell-level global view of all tissues and *CD276* expression sites is shown. **F-K** *CD276* expression levels of each cell type in adult cerebellum and fetal brain is shown. **L** and **M** *CD276* expression levels in each cell type in the brain were analyzed using different single-cell databases. **N** *CD276* expression levels in different cell types are shown. **O** and **R** Cell type distribution, and **P** and **S** *CD276* expression in basal cell carcinoma and breast cancer are shown. **Q** and **T** *CD276* expression differences in basal cell carcinoma and breast cancer are shown

The role of *CD276* in cancer was analyzed using pan-cancer samples from TCGA database and single-cell sequencing analyses. The research and design flow chart used in this study are shown in Fig. 1. First, the expression profile of *CD276* in cancer was analyzed using the CancerSEA database, and it was found that the expression level of *CD276* in various cancer types was high, including brain higher-grade glioma (HGG), ovarian serous cystadenocarcinoma (OV), glioma, GBM and breast invasive carcinoma (BRCA) (Fig. 2A). The HCA database may be used to exhaustively analyze the cell types of human embryos and adults at the single-cell level. Fig. 2B and D show the overall view at the single-cell level, whereas Fig. 2C and E show the overall view of brain tissue at the single-cell level. *CD276* was found to be strongly expressed in the tissues of the cerebellum, colorectum and embryo. We also analyzed the relative expression of *CD276* in adult cerebellum and fetal brain tissues, and the findings revealed that epithelial cell expression in the adult cerebellum was the strongest (Fig. 2F-H), whereas *CD276* was also highly expressed in astrocytes, GABAergic neurons, microglia, oligodendrocytes and stem cells in fetal brain tissues (Fig. 2I-K). In addition, the Tabula Muris database was used to explore cell clustering and the expression of *CD276* in cell subsets of the brain, and the results obtained revealed that *CD276* expression was mainly clustered in oligodendrocyte precursor cells, neurons and astrocytes of the cerebellum, Bergmann glial cells and brain pericytes, while *CD276* was also highly expressed in oligodendrocyte precursor cells (Fig. 2L-M), which was consistent with previous results. Subsequently, the monocellular immune module from the TIGER database was used to analyze *CD276* expression in various types of cancer and their corresponding immune cells. Fig. 2N clearly shows that the expression levels of *CD276* in basal cell carcinoma and breast cancer were high; the ensuing specific analysis revealed that the expression of *CD276* in fibroblasts in basal cell carcinoma was the highest, whereas its expression in melanocytes and fibroblasts in breast cancer was the strongest (Fig. 2O-T).

### *CD276* mRNA Expression Analysis in Human Pan-Cancer

**Fig. 3:**
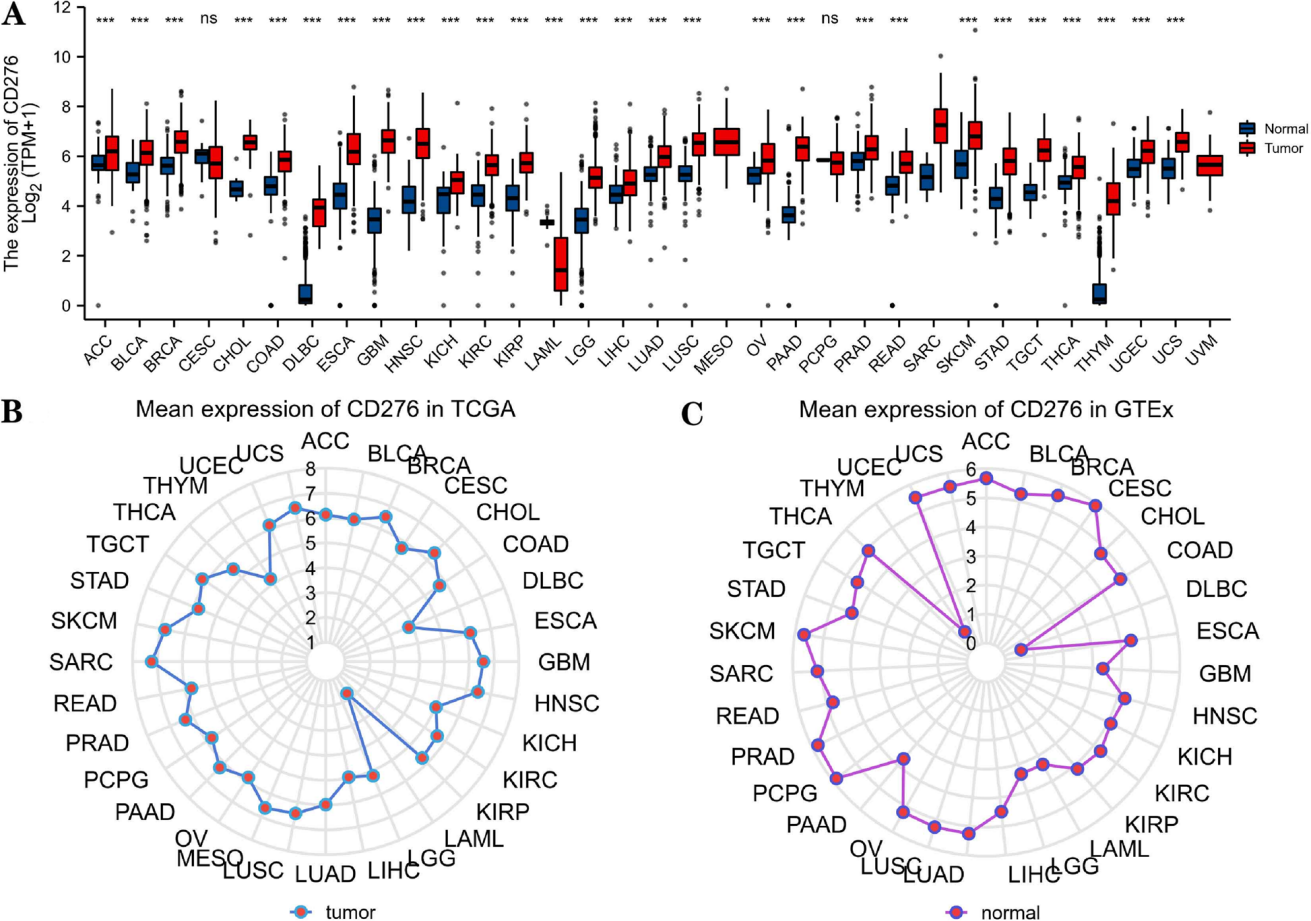
The expression of *CD276* in pan-cancer. **A** *CD276* expression among 33 cancer tissues in TCGA database and normal tissues in GTEx database is shown. **B** *CD276* mRNA expression levels in pan-cancers; the dotted positions in the figure represent the average expression level. **C** The mRNA expression levels of *CD276* in normal tissues in the GTEx database; the dotted positions represent the mean expression level of *CD276*. *P<0.05; **P<0.01; ***P<0.001; ns, no statistical significance

TCGA and GTEx datasets were then employed to evaluate the differences in *CD276* mRNA expression comparing between different types of cancer and normal tissues. The results showed that the *CD276* mRNA expression levels in cervical squamous cell carcinoma and endocervical adenocarcinoma (CESC), acute myeloid leukemia (LAML) and pheochromocytoma and paraganglioma (PCPG) were lower compared with that in normal tissues, among which the difference in expression was found to be statistically significant in the case of LAML. By contrast, *CD276* expression was higher in most other cancer types compared with that in normal tissues, including adrenocortical carcinoma (ACC), bladder urothelial carcinoma (BLCA), BRCA, cholangiocarcinoma (CHOL), colon adenocarcinoma (COAD) and GBM (Fig. 3A). In addition, *CD276* expression was found to be higher in sarcoma (SARC), skin cutaneous melanoma (SKCM), GBM and lung squamous cell carcinoma (LUSC), but lower in LAML, thymoma (THYM) and lymphoid neoplasm diffuse large B-cell lymphoma (DLBC) for tumor tissues in TCGA (Fig. 3B), whereas markedly higher expression levels of *CD276* were identified in the uterus, prostate and skin tissue for normal tissues in the GTEx cohort, with *CD276* expression being the lowest in the thymus and pancreas (Fig. 3C).

In order to further explore *CD276* expression for paired normal and tumor tissues, TCGA database was employed. This analysis revealed that *CD276* was highly expressed in almost all categories of tumors, including BLCA, BRCA, CHOL, COAD, esophageal carcinoma (ESCA), head and neck squamous cell carcinoma (HNSC), kidney chromophobe (KICH), kidney renal clear cell carcinoma (KIRC), kidney renal papillary cell carcinoma (KIRP), liver hepatocellular carcinoma (LIHC), lung adenocarcinoma (LUAD), LUSC, PRAD, stomach adenocarcinoma (STAD) and thyroid carcinoma (THCA) (Supplementary Fig. S1A-P), whereas *CD276* was lowly expressed in pancreatic ductal adenocarcinoma (PAAD) (Supplementary Fig. S1A).

### Expression of *CD276* in different cancers types based on individual cancer stages

We further assessed the association between *CD276* expression and the clinicopathological characteristics of the different tumor stages, and found that *CD276* expression was higher in more advanced individual cancer stages for almost all types of tumors, including ACC, BLCA, BRCA, CHOL, COAD, ESCA, HNSC, KICH, KIRC, KIRP, LIHC, LUAD, LUSC, rectum adenocarcinoma (READ), STAD, THCA, uterine corpus endometrial carcinoma (UCEC) and uveal melanoma (UVM) (Supplementary Fig. S2A-SM and S2P-ST). By contrast, *CD276* expression was markedly decreased in OV and PAAD (Supplementary Fig. S2N-SO). Furthermore, the expression of *CD276* was found to be similar in different tumor stages for certain types of cancers, and the differences were not statistically significant, including CESC, lymphoid neoplasm diffuse large B-cell lymphoma (DLBC), mesothelioma (MESO), SKCM, testicular germ cell tumors (TGCT) and uterine carcinosarcoma (UCS) (Supplementary Fig. S3).

### Genetic localization, alterations and DNA methylation of *CD276*

**Fig. 4:**
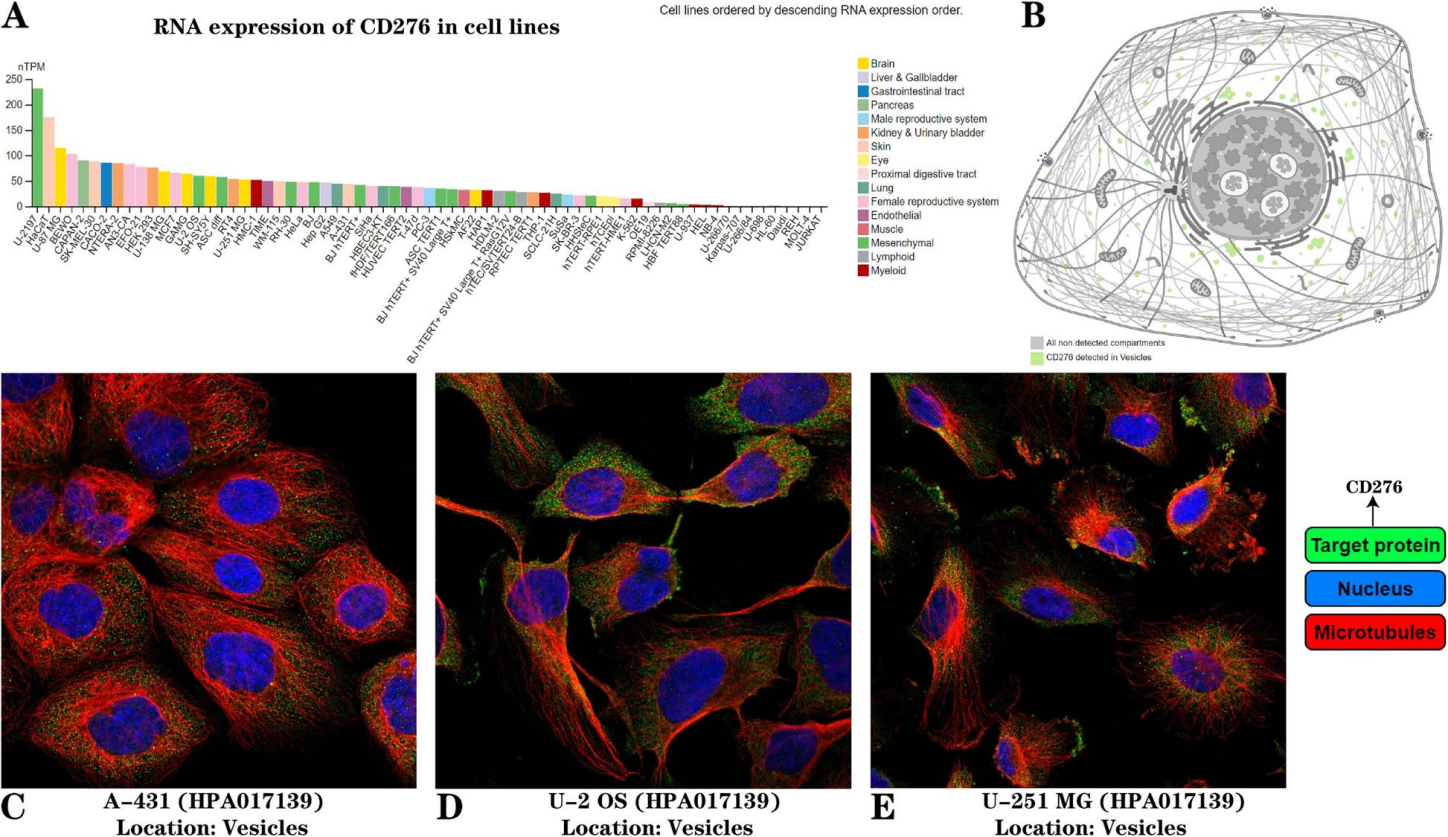
**A** Expression of *CD276* in tumor cell lines. **B** Subcellular localization of *CD276*. **C**-**E** Immunofluorescence analysis, showing *CD276* localization in three tumor cell lines, including A-431, U-2 OS and U-251 MG cells

The “Subcellular-RNA expression” section of the HPA database was employed to assess the RNA expression of *CD276* in different cell lines, and the results obtained revealed that *CD276* expression was the highest in three cell lines, specifically the U-2197, HaCaT and U-87 MG cell lines (i.e., mesenchymal, skin and brain cell types, respectively) (Fig. 4A). Subsequently, the “Subcellular-Human cells” section was explored, revealing that the predicted location of *CD276* was the intracellular membrane (Fig. 4B), which was as expected, considering that *CD276* is already known to be a potential immune checkpoint molecule. Furthermore, the distribution of *CD276* was evaluated within the nucleus, microtubules and endoplasmic reticulum, which confirmed that *CD276* was located in the vesicles in A-431, U-2 OS and U251 MG cell lines (Fig. 4C-E).

**Fig. 5:**
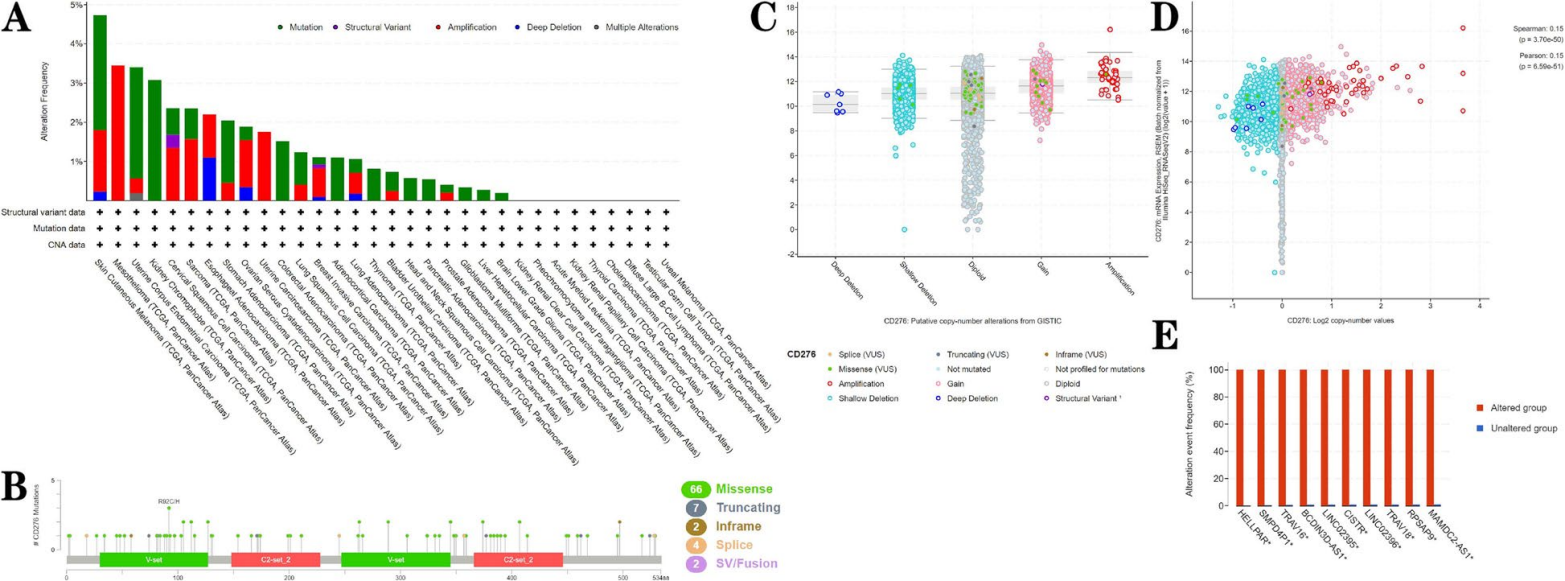
**A-D** Epigenetic alterations, types of gene mutations and copy numbers of *CD276* in different types of cancer were investigated. **E** The frequencies of several gene alterations that co-occur with *CD276* alterations are shown

Genetic and epigenetic alterations are known to influence variations in gene expression [46]. The cBioPortal database was then utilized to explore the categories and frequencies of genetic alterations of *CD276* for different types of cancer. The results obtained showed that mutations and amplifications of *CD276* were the leading types of genetic alterations [47, 48], especially for the SKCM, COAD and BRCA cancer types (Fig. 5A). With regard to *CD276* mutations, missense mutations were found to be the most common type (Fig. 5B). Copy-number values were significantly associated with shallow deletion, diploid and gain alterations of *CD276* (Fig. 5C and D). In addition, Fig. 5E shows that alterations in the frequencies of *HELLPAR, SMPD4P1, TRAV16, BCDIN3D-AS1, LINC02395, CISTR, LINC02396, TRAV18, RPSAP9* and *MAMDC2-AS1* occurred simultaneously with alterations of *CD276*.

DNA methylation is a form of epigenetic modification that regulates gene expression, and hypermethylation may contribute to the inactivation of tumor suppressor genes [49]. Hence, the associations between DNA methylation and *CD276* expression in different types of cancer were investigated, and the results obtained revealed that the DNA methylation level of *CD276* was negatively correlated with *CD276* expression in many types of cancer, including BLCA, BRCA, HNSC, KIRP, LUAD, LUSC and TGCT, whereas it was positively correlated with other cancer types, including PCPG. Moreover, it was found that the DNA methylation level of *CD276* was observably decreased in almost all types of cancers in the UALCAN database, including BLCA, LIHC, LUAD, LUSC, PCPG and TGCT (Supplementary Figs. S4A and E-2I), and these decreases were statistically significant. By contrast, the DNA methylation status was increased in a minority of cancer types, including BRCA, HNSC and KIRP (Supplementary Fig. S4B-D). Taken together, these results suggested that *CD276*, as an immunosuppressor gene, is hypomethylated in the majority of cancer types.

**Fig. 6:**
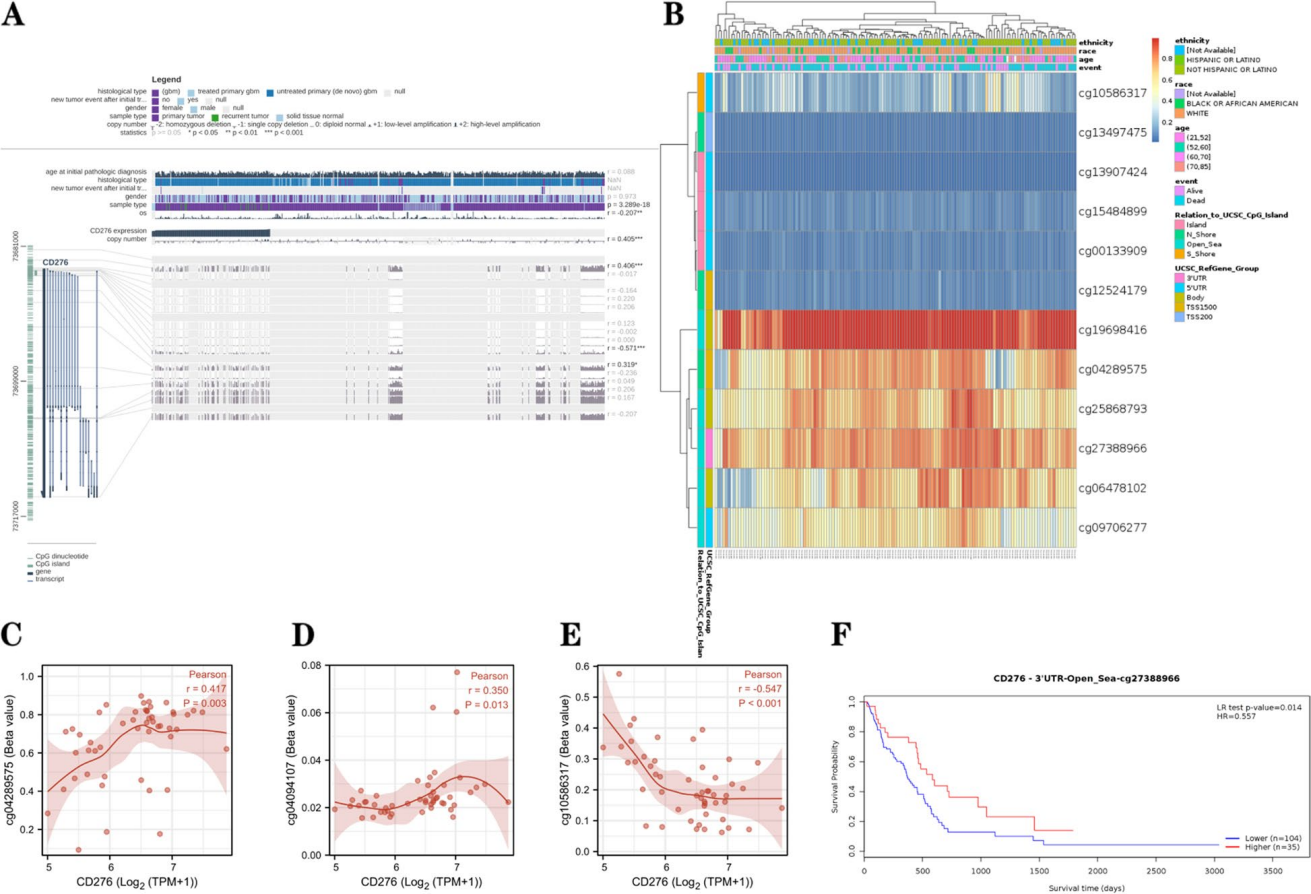
DNA methylation of *CD276*. **A** Methylation sites of the *CD276* DNA sequence associated with *CD276* expression were identified using the MEXPRESS database; *CD276* expression is shown by the blue line on the left side, whereas Pearson’s correlation coefficients for the methylation sites are shown on the right side. **B** Visualization of the DNA methylation level and *CD276* expression. **C-E** Expression levels of *CD276* at different methylation sites in GBM are shown. **F** The Kaplan-Meier survival plots of promoter methylation of *CD276* in patients with GBM are presented

Furthermore, we found that five DNA methylation sites (cg10586317, cg13497475, cg00133909, cg02161084 and cg27388966) of *CD276* in the DNA sequence were highly expressed, whereas the methylation levels in other probe regions (cg10586317, cg13497475, cg13907424, cg15484899, cg00133909 and cg12524179) were low (Fig. 6A). As shown in Fig. 6B, for *CD276*, low methylation levels were found at most DNA methylation sites. The effects of different methylation sites on *CD276* expression were then analyzed in TCGA-GBM database, which revealed that cg04289575 and cg04094107 were positively correlated with *CD276* expression, whereas cg10586317 was negatively correlated with *CD276* expression (Fig. 6C-E). The MethSurv database was subsequently investigated to assess the effect of the GBM hypomethylation level and *CD276* expression on prognosis, which showed that cg27388966 located on the CpG island was associated with poor prognosis (Fig. 6F).

### *CD276* Protein Expression and the PPI Network Analysis

**Fig. 7:**
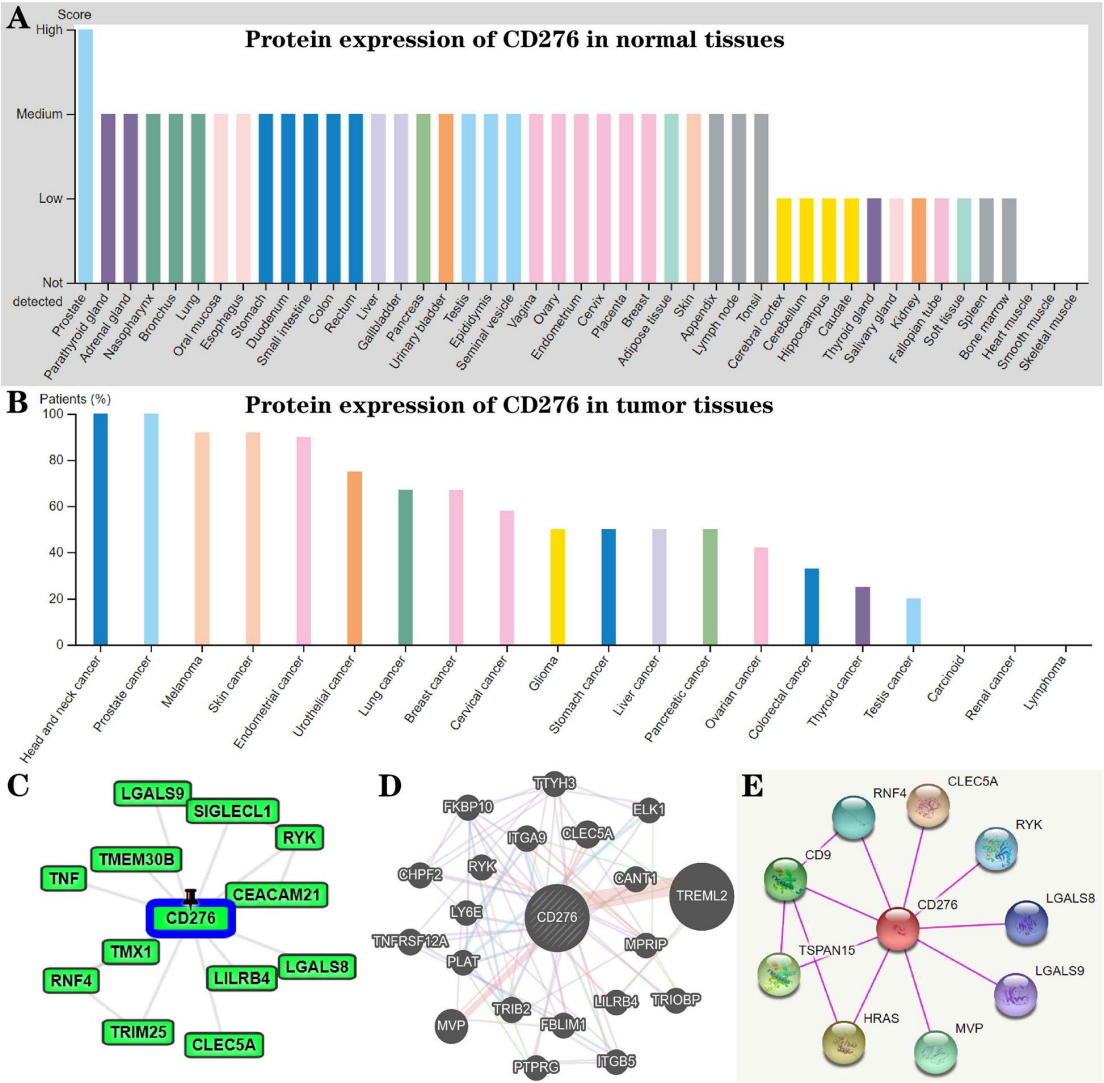
Pan-cancer *CD276* protein expression in different types of cancers. **A** and **B** The *CD276* expression levels in normal human tissues are shown in (**A**), whereas the expression levels of *CD276* in different types of cancer are shown in (**B**). **C-E** PPI network was constructed using the GPS-PROT, GeneMANIA and STRING databases, and a series of proteins closely associated with *CD276* were identified

In order to detect the *CD276* protein expression profiles in human normal and cancer tissues, the HPA dataset was employed. The results indicated that the protein expression level of *CD276* was high in the prostate, whereas it was low in heart muscle, smooth muscle and skeletal muscle (Fig. 7A). In addition, the protein expression level of *CD276* was found to be the highest in head and neck cancer and prostate cancer, whereas it was lowest in carcinoid tumors, renal cancer and lymphoma (Fig. 7B). The corresponding immunohistochemical results revealed the protein expression levels of *CD276* (Supplementary Fig. S5).

Furthermore, the GPS-Prot, GeneMANIA and STRING databases were used to construct the PPI network, and these data revealed that six genes were closely associated with *CD276*, including *RYK* (receptor protein tyrosine kinases), *CLEC5A* [spleen tyrosine kinase (syk)-coupled C-type lectins], *RNF4* (recombinant human ring finger protein 4), *LGALS8* (galectin-8 gene) and *LGALS9* (galectin-9 gene) (Fig. 7C-E). Subsequently, the intersection of these three groups of genes was used for functional enrichment analysis, including GO and KEGG analysis, employing the DAVID database. The top 3 biological processes (BP) enriched terms were ‘interferon-gamma production’, ‘regulation of interferon-gamma production’ and ‘positive regulation of interferon-gamma production’. The top 3 cellular components (CC) enriched terms were ‘cell-substrate junction’, ‘cell-substrate adherens junction’ and ‘focal adhesion’. Finally, the top two molecular functions (MF) enriched terms were ‘cell adhesion molecule binding’ and ‘integrin binding’. The top 3 KEGG enriched terms were ‘hypertrophic cardiomyopathy’, ‘proteoglycans in cancer’ and ‘focal adhesion’ (Supplementary Fig. S6 and Table 1).

**Table 1:** GO and KEGG enrichment analysis of *CD276* and their interactors

Ontology	ID	Description	GeneRatio	Genes	pvalue	p.adjust	qvalue
BP	GO:0032729	positive regulation of interferon-gamma production	4/30	HRAS, LGALS9, TNF, *CD276*	3.43e-06	0.004	0.003
BP	GO:0032649	regulation of interferon-gamma production	4/30	HRAS, LGALS9, TNF, *CD276*	1.98e-05	0.010	0.007
BP	GO:0032609	interferon-gamma production	4/30	HRAS, LGALS9, TNF, *CD276*	3.09e-05	0.010	0.007
CC	GO:0005925	focal adhesion	5/33	CD9, ITGB5, TRIOBP, MPRIP, FBLIM1	5.27e-04	0.014	0.012
CC	GO:0005924	cell-substrate adherens junction	5/33	CD9, ITGB5, TRIOBP, MPRIP, FBLIM1	5.45e-04	0.014	0.012
CC	GO:0030055	cell-substrate junction	5/33	CD9, ITGB5, TRIOBP, MPRIP, FBLIM1	5.70e-04	0.014	0.012
MF	GO:0005178	integrin binding	3/31	CD9, ITGB5, LGALS8	0.002	0.091	0.076
MF	GO:0050839	cell adhesion molecule binding	5/31	CD9, ITGB5, LGALS8, TRIM25, MPRIP	0.002	0.091	0.076
KEGG	hsa04510	Focal adhesion	4/14	ELK1, HRAS, ITGA9, ITGB5	3.06e-04	0.018	0.013
KEGG	hsa05205	Proteoglycans in cancer	4/14	ELK1, HRAS, ITGB5, TNF	3.30e-04	0.018	0.013
KEGG	hsa05410	Hypertrophic cardiomyopathy	3/14	ITGA9, ITGB5, TNF	4.46e-04	0.018	0.013

### The prognostic significance of *CD276* expression in cancer patients

**Fig. 8:**
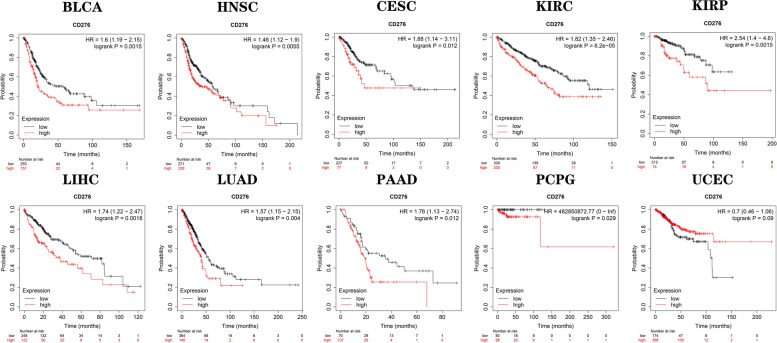
Prognostic significance of *CD276* in different types of cancer. Kaplan-Meier overall survival curves were plotted to compare low and high expression of *CD276* in the indicated cancer categories from TCGA database

**Fig. 9:**
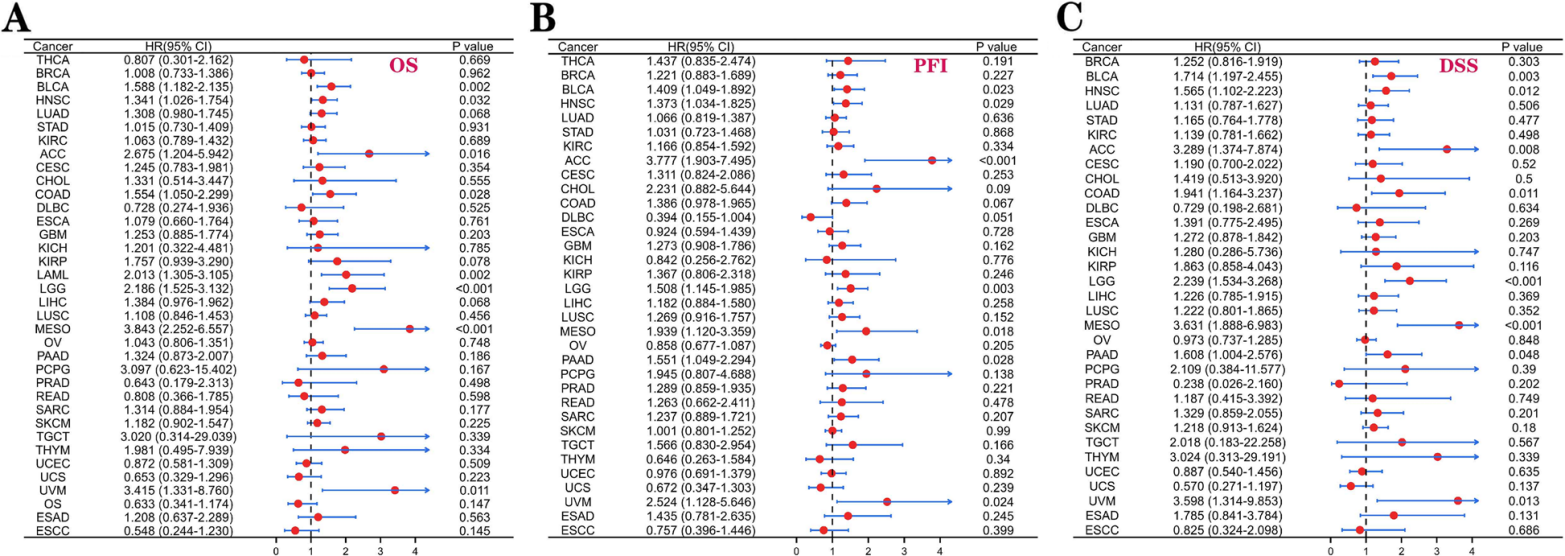
Univariate Cox regression analysis of *CD276*. **A-C** The forest plot in **A** shows the correlation between *CD276* expression in different types of cancer and the Cox regression model of OS rates. **B** shows the univariate Cox regression results of *CD276* in terms of the PFI rates of 33 types of cancer in TCGA database. **C** shows the univariate Cox regression results of *CD276* for the DSS rates of the various types of cancer

Subsequently, we researched the prognostic value of *CD276* in cancer patients. The Kaplan-Meier database was employed, and the results obtained showed that *CD276* expression was lower in the BLCA, HNSC, CESC, KIRC, KIRP, LIHC, LUAD, PAAD and PCPG cancer types, indicating was *CD276* presented a hazard for patients (Fig. 8). By contrast, *CD276* was found to act as a protective factor in patients with UCEC (Fig. 8). Subsequently, univariate Cox regression analysis was performed, and the OS rates obtained revealed that *CD276* served as a risk element in patients with BLCA, HNSC, ACC, COAD, LAML, LGG, MESO and UVM, whereas it was found not to be a protective factor for the various types of cancer (Fig. 9A). The PFI analysis showed that *CD276* acts as a hazardous element in patients with BLCA, HNSC, ACC, LGG, MESO, PAAD and UVM, whereas it serves as a protective element for patients with DLBC (Fig. 9B). Finally, the DSS analysis revealed that *CD276* serves as a hazardous element in numerous types of cancer, including BLCA, HNSC, ACC, COAD, LGG, MESO, PAAD and UVM (Fig. 9C).

### *CD276*-associated signaling pathways identified by GSEA in different types of cancer

**Fig. 10:**
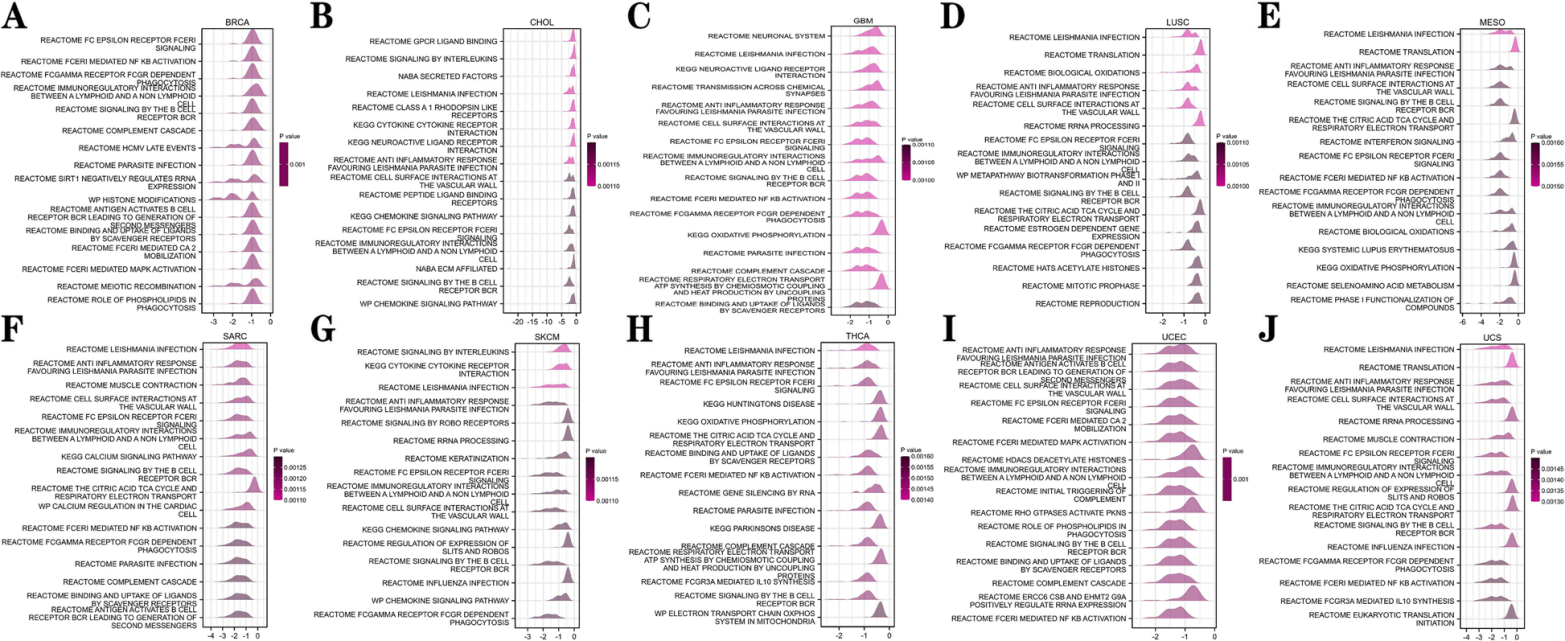
Merged enrichment plots for *CD276* on the basis of GSEA in pan-cancer. **A**-**J** The signaling pathways associated with *CD276* according to GO, KEGG and Reactome analyses, including those involved in cancer immunoregulation

The molecular mechanisms and signaling pathways that may be associated with *CD276* were then investigated by employing GSEA of cancers from TCGA cohort. The results obtained showed that *CD276* was strongly correlated with different immune-associated functions, including immunoregulatory interactions between lymphoid and a non-lymphoid cell and signaling mediated by the B-cell receptor (BCR) in BRCA, CHOL, GBM, LUSC, MESO, SARC, SKCM, THCA, UCEC and UCS (Fig. 10). In addition, *CD276* was found to be associated with nonspecific immunomodulatory effects, such as neutrophil degranulation and signaling by interleukins, in certain types of cancer, including KICH, LAML, LGG, LIHC, PCPG, READ, STAD and UVM (Supplementary Fig. S7). In conclusion, *CD276* was confirmed to fulfill a crucial role in mediating the inflammatory response and regulating the tumor immune microenvironment.

### Association Between *CD276* and Immune Cell Infiltration in Pan-Cancer

**Fig. 11:**
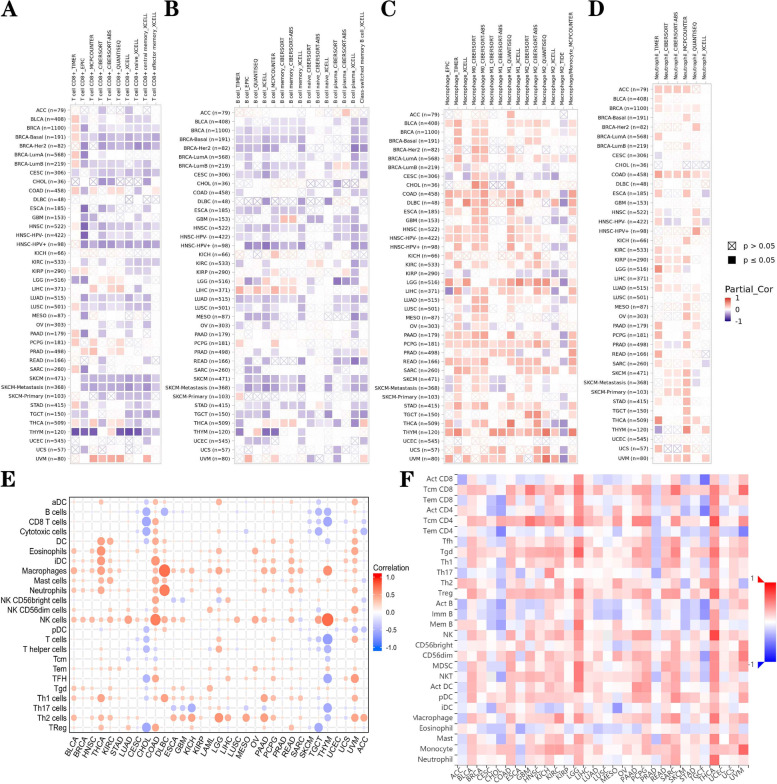
Correlation of *CD276* expression with immune cell infiltration in the different types of cancer. The correlation between *CD276* and infiltration of **A** CD8+ T cells and **B** B cells are shown. **C** and **D** The correlation between *CD276* and infiltration levels of macrophages and neutrophils, respectively, are shown. **E** The correlation between *CD276* and immune cell infiltration **F** shows the correlation between *CD276* and the levels of immune cell infiltration, as assessed using the TISDB database

Since immune infiltrating cells are closely associated with the occurrence and development of cancer, three immune cell infiltration analysis methods were used to investigate the correlation between *CD276* expression and immune cell infiltration. The TIMER2 dataset revealed that *CD276* was negatively correlated with the infiltration levels of CD8+ T cells and B cells, whereas *CD276* expression was positively correlated with macrophages and neutrophils in TCGA in pan-cancer (Fig. 11A-D) [50]. Based on the CIBERSOFT dataset, *CD276* was found to be negatively correlated with CD8+ T cells, B cells and T cells, whereas *CD276* expression was positively correlated with macrophages and neutrophils in numerous types of cancer (Fig. 11E). Finally, using the TISDB database, it was found that *CD276* expression was negatively associated with Act CD8 cells, Act B cells and Tem CD4 cells in most types of cancer, but positively correlated with macrophages and neutrophils (Fig. 11F). Generally speaking, *CD276* may inhibit T cell infiltration, which may account for its role as a risk element in most types of cancer.

### Association between *CD276* and immunoregulation-associated genes

**Fig. 12:**
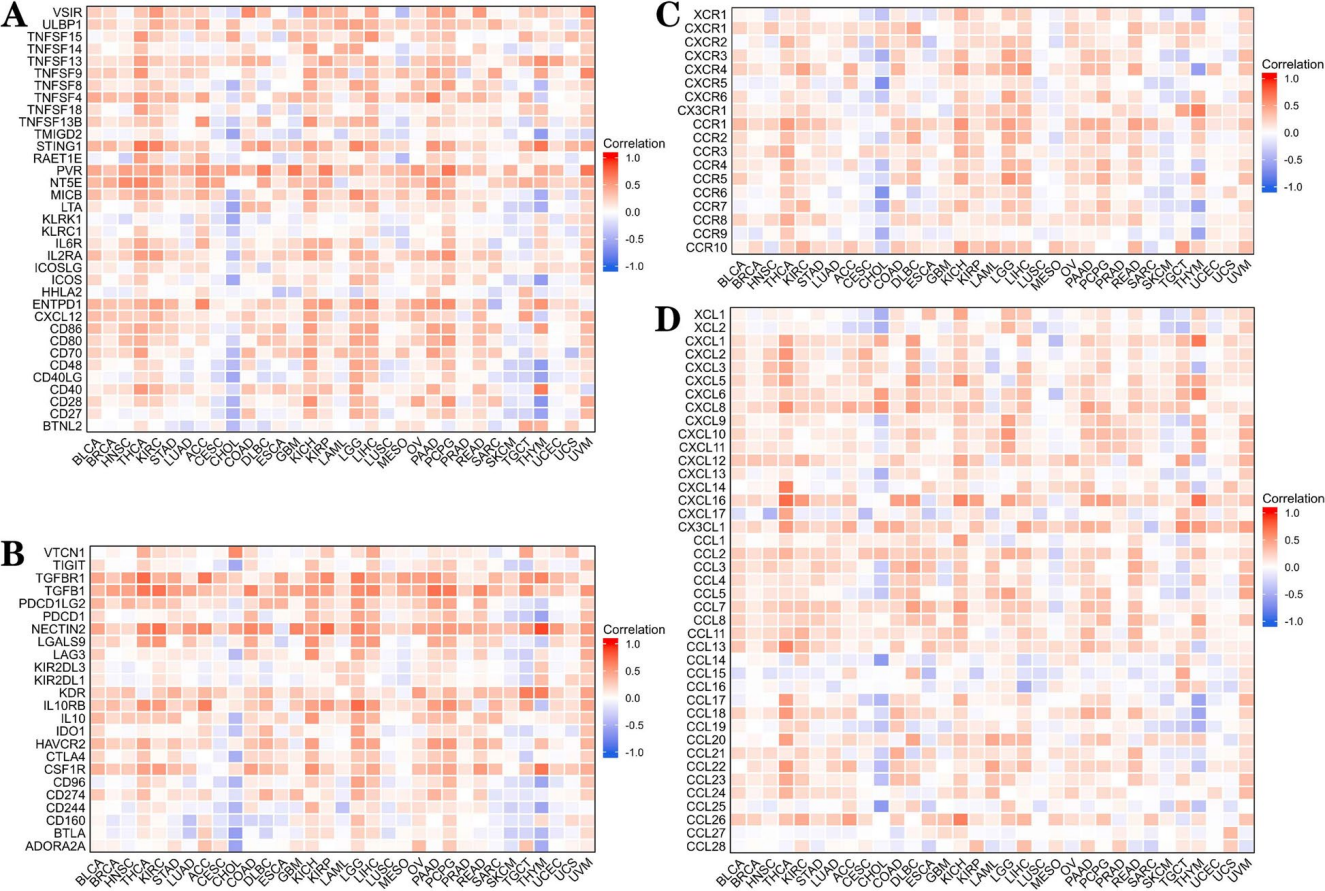
The correlation between *CD276* and immunoregulation-associated genes. **A** illustrates the correlation between *CD276* expression and immune-activating genes. B shows the correlation between *CD276* expression and immunosuppression-associated genes. **C** highlights the correlation between *CD276* expression and chemokine receptors, whereas **D** shows the correlation between *CD276* expression and a variety of chemokines

Since immune checkpoint genes provide important targets for cancer immunotherapy, the associations among immune-activating genes, immune checkpoint genes, chemokine genes and chemokine receptor genes and *CD276* expression were subsequently analyzed. The results revealed that *CD276* was significantly associated with most immunostimulatory genes, including *PVR* (poliovirus receptor), *ENTPD1* (ectonucleoside triphosphate diphosphohydrolase 1) and *NT5E* (ecto-5'-nucleotidase), in PCPG, KICH, LGG and THCA (Fig. 12A). Moreover, we found that *CD276* expression was positively correlated with immune checkpoint genes in the LGG, LIHC, PCPG and THCA cancer types, including *TGFBR1* [transforming growth factor beta (TGF-beta) receptor 1], *TGFB1* (transforming growth factor beta 1), *NECTIN2* (poliovirus receptor-related protein 2), *IL10RB* (interleukin 10 receptor beta) and *CSF1R* (colony-stimulating factor-1 receptor) (Fig. 12B). Furthermore, *CD276* expression was significantly correlated with chemokine receptor genes, including *CCR1* [chemokine (C-C motif) receptor 1], in the KICH, LGG, LIHC and THCA cancer types (Fig. 12C). Finally, the expression level of *CD276* was positively associated with chemokine genes, including *CCL7* [Chemokine (C-C motif) ligand 7], *CXCL16* (CXC chemokine ligand 16) and *CXCL8* (CXC chemokine ligand 8), in THCA, KICH, PAAD and UVM (Fig. 12D). Interestingly, *CD276* expression was found to be negatively correlated with most types of immune-activating genes, immune checkpoint genes, chemokine genes and chemokine receptor genes in CHOL (Fig. 12A-D). In addition, the TISDB database was utilized to explore the associations among *CD276* expression and immunoinhibitory genes, immunostimulatory genes, chemokines and chemokine receptors, and this analysis produced similar results (Supplementary Fig. S8). Collectively, these results revealed the potential role of *CD276* as a tumor immune checkpoint molecule, although further in vivo experiments and clinical trials are required to verify the antitumor activity and immune checkpoint effects of *CD276*.

### *CD276* expression and its prognostic value in GBM

In order to further explore the expression of *CD276* in GBM, TCGA and GEO databases were used to reveal that the mRNA expression level of *CD276* in GBM was significantly higher compared with that in the normal control group (Supplementary Fig. S9A and B). The CPTAC module of the UALCAN database was used to analyze the protein expression of *CD276*, which showed that the protein expression level of *CD276* in GBM was also significantly higher compared with that in normal controls (Supplementary Fig. S9C). In addition, we found that patients with GBM and with a high expression level of *CD276* had lower OS rates, which may be associated with poor prognosis of patients (Supplementary Fig. S9D and E).

### Analysis of *CD276* expression and the mutational landscape

**Fig. 13:**
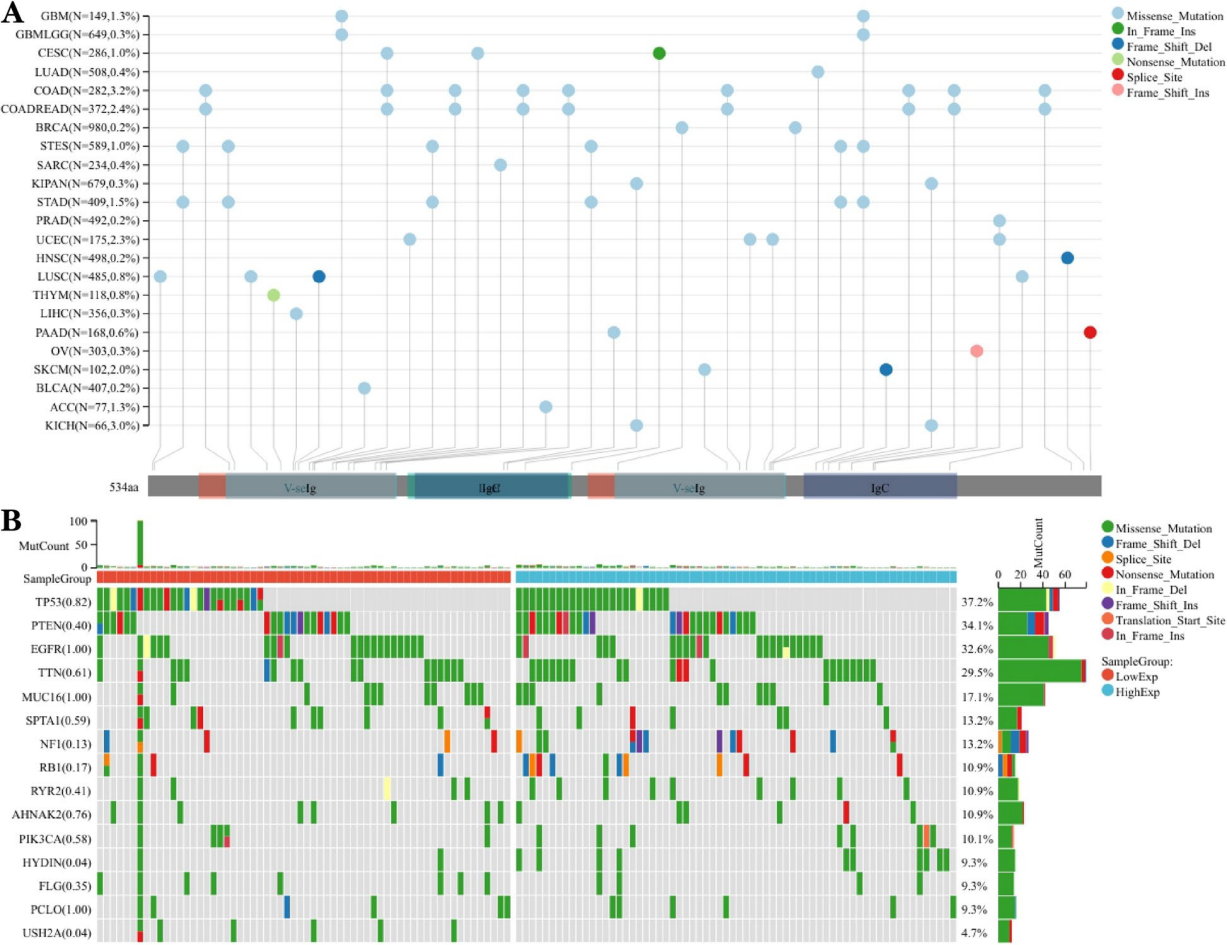
Mutational landscape analysis. **A** Pan-cancer analysis of *CD276* gene mutation types in various types of cancers. **B** The mutation types and mutant genes of the high- and low-expression groups of *CD276* in GBM were analyzed

Sangerbox 3.0 (http://sangerbox.com/home.html) was used to analyze the mutational landscape of *CD276* in pan-cancer, and the associations among different *CD276* expression levels and the gene mutational landscape in GBM. Missense mutations were found to occur in the vast majority of cancers, including GBM (Fig. 13A). Subsequently, the gene mutation landscapes of the low and the high *CD276* expression groups in GBM were further analyzed. The most common mutation type was found to be missense mutations, and the most common mutated genes were *TP53, PTEN, EGFR* and *TTN* (Fig. 13B).

### The *CD276* Co-Expression Network in GBM

**Fig. 14:**
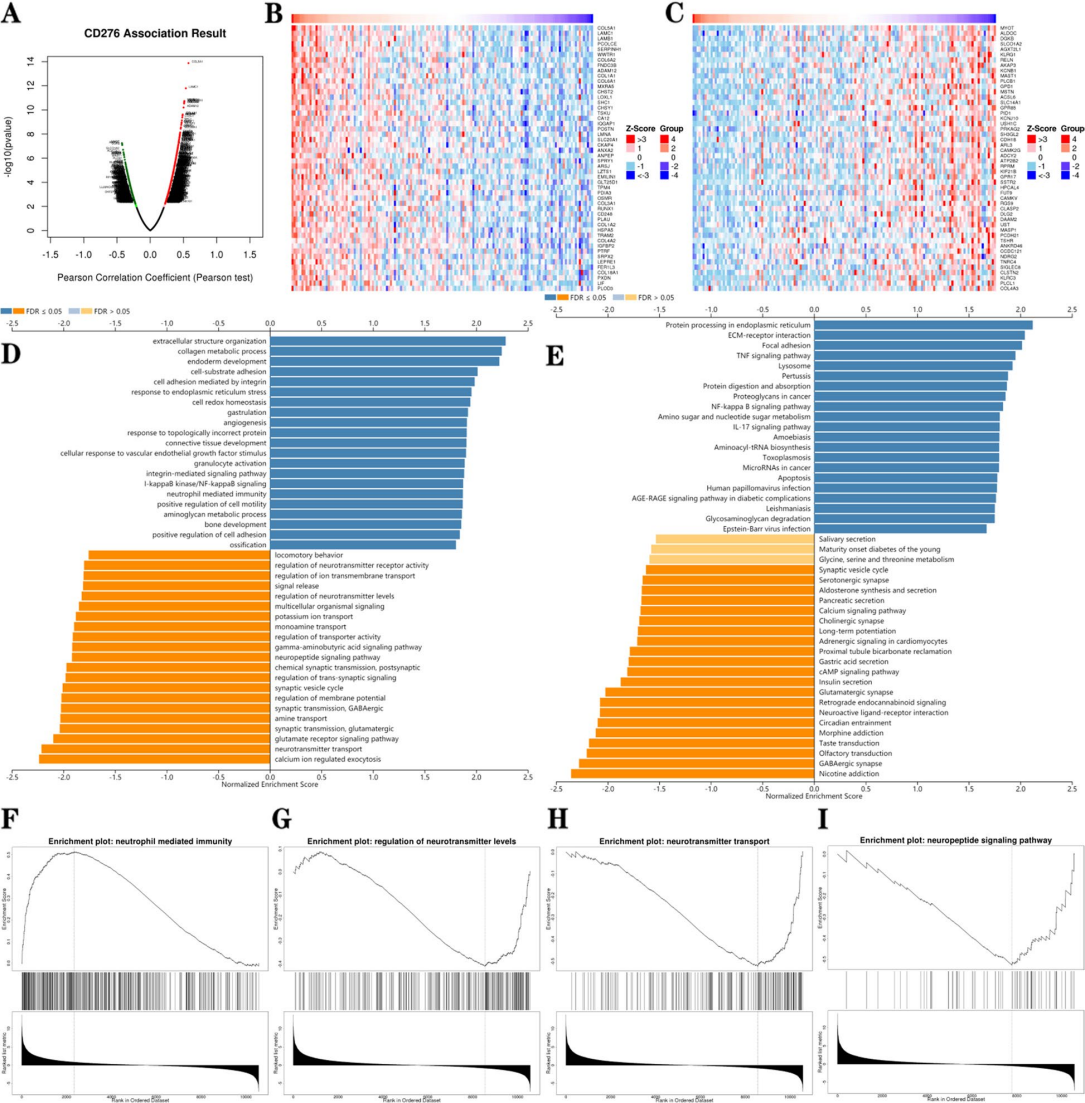
Enrichment analysis of co-expressed genes with *CD276* in glioblastoma using the LinkedOmics database. **A** Positively and negatively correlated genes of *CD276* were differentiated using Pearson’s test in the LUAD cohort. **B** and **C** The heatmaps show the top 50 genes that were positively and negatively correlated with *CD276*, respectively. The positively correlated genes are shown in red, whereas negatively correlated genes are shown in blue. **D** and **E** GO and KEGG pathway analyses of *CD276* in glioblastoma. **F-I** Enrichment analysis, showing that the expression of *CD276* in glioblastoma was positively correlated with neutrophil-mediated immunity, whereas *CD276* expression was negatively associated with regulation of neurotransmitter levels, neurotransmitter transport and neuropeptide signaling pathways

The LinkedOmics database was then used to explore the biological functions and co-expression patterns of *CD276* in GBM. Fig. 14A shows all the genes that were positively and negatively correlated with *CD276*, whereas Fig. 14B and C show the top 50 genes that were positively and negatively correlated with *CD276*, respectively. GO analysis showed that the *CD276* co-expressed genes were mainly involved in extracellular structure organization, collagen metabolic processes, endoderm development, locomotory behavior, regulation of neurotransmitter receptor activity, regulation of ion transmembrane transport, and so on (Fig. 14D). KEGG pathway analysis revealed that *CD276* co-expressed genes were mainly enriched in protein processing in the endoplasmic reticulum, extracellular matrix (ECM)-receptor interaction, focal adhesion, the *TNF* signaling pathway, salivary secretion, maturity onset diabetes of the young and glycine, serine and threonine metabolism (Fig. 14E). Notably, the expression of *CD276* in GBM was positively correlated with neutrophil-mediated immunity, whereas it was negatively associated with regulation of neurotransmitter levels, neurotransmitter transport and neuropeptide signaling pathways (Fig. 14F-I). Taken together, these results suggested that *CD276* expression networks have a broad impact on the immune activation of GBM.

### Functions and treatment of *CD276* in GBM

**Fig. 15:**
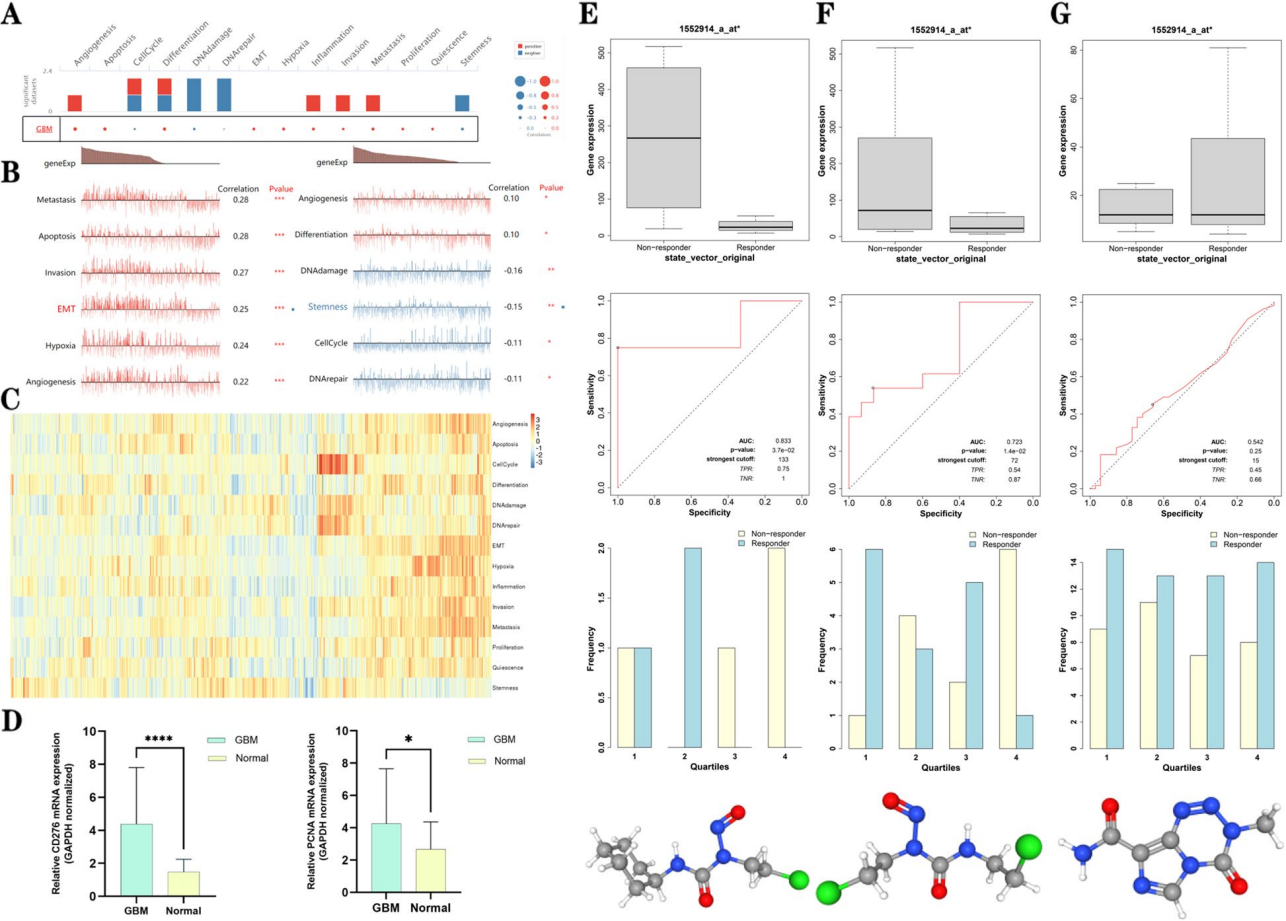
*CD276* expression is associated with the functions and treatment of GBM. **A** Single-cell analysis showed that *CD276* is involved in regulating angiogenesis, proliferation, DNA damage and repair, EMT, inflammation, invasion and metastasis. **B** and **C** Data from the CancerSEA database showed that *CD276* was correlated with various functions. **D** RT-qPCR was used to analyze *CD276* and *PCNA* mRNA expression in patients with GBM compared with that in normal tissues. **E** and **G** The ROC plotter database was used to explore the relationship between *CD276* expression and responses to chemotherapy in patients with GBM

In order to explore the functions of *CD276* in GBM, single-cell analysis was performed using the CancerSEA database. The results obtained showed that *CD276* could positively regulate GBM cell proliferation, invasion, migration, angiogenesis, epithelial-mesenchymal transition (EMT), apoptosis and inflammation, whereas it negatively regulated GBM cell DNA damage and repair, cell cycle and tumor cell stemness (Fig. 15A-C). In order to increase the reliability of the single-cell analysis, RT-qPCR analysis was subsequently performed to analyze the expression of *CD276* in patients with GBM. By analyzing 18 human tissue samples, it was found that the expression levels of *CD276* and *PCNA* in GBM tissues were significantly higher compared with those in normal tissues (Fig. 15D), findings that were also consistent with the results reported in Fig. 3A, thereby providing guidance for our future research in this direction.

In addition, the ROC Plotter database was used to explore the influence of the *CD276* expression level on chemotherapy drug sensitivity of patients with GBM. The results obtained showed that patients with GBM with high expression levels of *CD276* were resistant to lomustine (Fig. 15E) and nitrosourea (Fig. 15F), whereas no statistical significances were identified with regard to their sensitivity to temozolomide (Fig. 15G).

## Discussion

It is well established that cancer has become the most serious disease threatening human health and life throughout the world, and timely diagnosis and treatment of cancer patients is crucially important [1]. Although cancer can be effectively combatted through the use of surgery, radiotherapy and chemotherapy, the OS rate of patients with cancer remains poor. In recent years, immunotherapy has improved the status of cancer therapeutic methods, and immune checkpoint blockade therapy has become one of the most promising immunotherapy methods [51]. Common immune checkpoints include *PD-L1, PD-1* and *CTLA4* [52]. As an important immune checkpoint molecule, *CD276* has a vital role in inhibiting T cell function, and because *CD276* is highly expressed in most tumor cells, it may be a putative target for cancer immunotherapy in the future [7]. In the present study, we used single-cell analysis and bioinformatics methods to investigate *CD276* expression patterns, single-cell levels in human tissues, expression levels in cell subpopulations, and gene expression in different cell types in different brain tissues, as well as the expression of *CD276* in various types of cancer and its impact on patient prognosis, epigenetic alterations, DNA methylation, GSEA analysis and immune cell infiltration levels.

The Human Cell Landscape website was used for single-cell RNA sequencing to determine the cell type composition of major human organs, and to construct a basic scheme for the human cell landscape. Our analysis revealed that *CD276* was highly expressed in cerebellar, colorectal and embryonic tissues. The relative expression levels of *CD276* in adult and fetal brain tissue were also found to be different, with the strongest expression found in adult epithelial cells, and high expression levels were also identified in fetal astrocytes, neurons, microglia, oligodendrocytes and stem cells. The Tabula Muris website is a compendium of single-cell transcriptome data from the model organism Mus musculus, containing nearly 100000 cells from 20 organs and tissues. We explored the brain cell clustering and expression patterns of *CD276* in cell subsets using the Tabula Muris database, and found that *CD276* was mainly accumulated in oligodendrocyte precursor cells, neural cells and brain astrocytes, a finding that was consistent with previous results. TIGER is a web-accessible portal for integrative analysis of the gene expression data associated with tumor immunology. TIGER contains bulk transcriptome data for 1,508 tumor samples with immunotherapy clinical data and 11,057 tumor/normal samples from TCGA, and single-cell transcriptome data for 2,116,945 cells of 655 samples, among which 119,039 cells of 63 samples have immunotherapy clinical data. We explored the single-cell immunity module in the TIGER database to analyze the expression of *CD276* in various types of cancer and their corresponding immune cells, and found that the expression level of *CD276* was higher in basal cell carcinoma and breast cancer; moreover, the expression level of *CD276* in fibroblasts was the highest in basal cell carcinoma, and the expression level of *CD276* was also strong in melanocytes and fibroblasts in breast cancer.

A comprehensive evaluation of *CD276* expression and prognosis in 33 types of cancer showed that *CD276* expression levels were dysregulated in most types of cancer, a finding that was in line with a previous study [7]. *CD276* expression in different tumors and the corresponding normal tissues was investigated on the basis of TCGA and GTEx datasets, and we discovered that *CD276* was highly expressed in 30 types of cancer, including ACC, BLCA and BRCA, but expressed at low levels in CESC, LAML and PCPG. In addition, we found that the protein expression level of *CD276* was highest in HNSC and PRAD, and lowest in carcinoid tumors, renal cancer and lymphoma. A previous study [53], through immunohistochemical detection analysis, showed that *CD276* was located in the cell membrane, cytoplasm, nucleus and vascular system. In patients with colorectal cancer, *CD276* was expressed in the cell membrane/cytoplasm in 86% of the patients, followed by the stroma, whereas the expression level was low in the nucleus [53]. Our study found that *CD276* was mainly found in the intracellular membrane of patients with cancer, although it was also highly expressed in the vesicles of the A-431, U-2 OS, and U251 MG cell lines. Furthermore, we established the PPI network of *CD276*, and discovered that *CD276* was closely associated with the proteins encoded by *RYK, CLEC5A, RNF4, MVP, LGALS8* and *LGALS9.*

In the present study, we also investigated the pathological and clinical significance of *CD276* in different types of cancers in TCGA, and *CD276* expression at the different cancer stages. Moreover, we analyzed the relationship between *CD276* expression and the OS, DSS and PFI rates in different cancer types. The Kaplan-Meier OS analysis showed that a high expression level of *CD276* is a risk element for the majority of cancer types, although it appears to be a protective factor for UCEC. For the DSS and PFI rates, we used univariate Cox regression analysis to show that a high expression level of *CD276* correlated with low survival in the majority of cancer types, including BRCA, ACC, COAD, LGG, MESO and UVM, whereas a high expression level of *CD276* appears to be a protective element for DLBC. Overall, these findings indicated that high *CD276* expression is a risk element for patients with cancer, and may predict poor survival and prognosis.

The above analysis revealed that *CD276* is highly expressed in most types of cancer, and is often associated with poor prognosis in patients. A previous study showed that *CD276* is able to induce antigen-presenting cells and has a crucial role in inhibiting T-cell function, suggesting that it is a promising target for cancer immunotherapy [7]. In order to analyze the molecular mechanism of *CD276* expression in tumorigenesis from the perspective of bioinformatics, and to investigate whether it has a connection with immune-associated signaling pathways, GSEA analysis of *CD276* in cancer was performed. The results obtained showed that *CD276* was highly correlated with immunoregulatory interactions between lymphoid and a non-lymphoid cell and signaling medicated by the B-cell receptor BCR in many types of cancer, including BRCA, CHOL, GBM, LUSC, MESO, SARC, SKCM, THCA, UCEC and UCS.

We obtained tumor immune cell infiltration results by utilizing the TIMER2, CIBERSOFT and TISDB databases, and found that CD8+ T cells and B cells were negatively correlated with the expression of *CD276* in most types of cancer. Given that cytotoxic CD8+ T cells are killer cells in the T lymphocyte population [54], this could explain the main tumor-promoting role of *CD276* in cancer. In addition, the expression of *CD276* was found to be positively associated with macrophages and neutrophils in TCGA pan-cancer, which suggested that *CD276* may be associated with macrophage polarization.

The occurrence and development of tumors cannot be separated from the regulation of the tumor microenvironment (TME), and the TME has attracted an increasing amount of attention in tumor immunotherapy. Cancers can evade the immune response and influence immunotherapy by means of immune checkpoint genes, such as *PD-L1* (programmed death-ligand 1), *PD-1* and *CTLA-4*. Therefore, identifying novel immunotherapy biomarkers is a critical aspect of tumor immunotherapy [55]. In the present study, our analysis has shown that *CD276* expression was positively correlated with a number of immune checkpoint genes, including *TGFBR1, TGFB1, NECTIN2, IL10RB* and *CSF1R*, in most types of cancer. In general, patients with cancer lose their T-cell functions, and have declining numbers of T-cells. We discovered that *CD276* expression was positively correlated with exhausted T-cell marker genes and immune checkpoint genes in tumors, including *CTLA4, PDCDD1LG2, KDR, HAVCR2, TGFBR1, TGFB1, NECTIN2, IL10RB* and *CSF1R*. However, further in vitro and in vivo studies are required to evaluate the usefulness of monoclonal antibodies against *CD276* for future therapeutic strategies.

Single-cell analysis revealed that *CD276* was highly expressed in adult cerebellum and fetal brain tissue. Further functional analysis of GBM showed that high expression levels of *CD276* were associated with the proliferation, invasion, migration and angiogenesis of tumor cells. RT-qPCR analysis subsequently confirmed the high proliferation level of GBM cells through the mRNA expression level of *PCNA*. In addition, the expression of *CD276* was found to be correlated with the sensitivity of GBM to chemotherapy drugs. Patients with GBM with high expression levels of *CD276* were resistant to lomustine and nitrosourea, whereas no sensitivity was identified with temozolomide treatment.

In general, our comprehensive evaluation of *CD276* has provided ample evidence to suggest that *CD276* may act as a potential prognostic biomarker and immunotherapy target. However, although information was utilized from different databases for systematic bioinformatics analysis, there were still a number of limitations associated with the present study. First, we used different databases and analytical methods, and several deviations occurred when comparing the results from certain studies; therefore, further in vivo and in vitro experiments are required to confirm the results. In addition, although we have explored the possible function and value of *CD276* in numerous types of cancers, these aspects need to be further verified. Finally, monoclonal antibodies against *CD276* are currently in phase I clinical trials, and further optimization of cancer immunotherapy approaches are required in humans.

## Conclusion

In conclusion, the present study has shown that *CD276* is expressed differently in different cancers and at different stages of cancer development, suggesting that *CD276* may exert different roles in different cancers, although, overall, high expression of *CD276* in the majority of different types of cancer is associated with poor patient prognosis, poor survival and poor clinical outcomes. For GBM, the expression level of *CD276* in adult cerebellum and fetal brain tissue may be relatively higher, and this high expression of *CD276* may be associated with GBM tissue proliferation, migration, invasion, EMT and angiogenesis, although it was negatively correlated with DNA damage and repair. In addition, patients with GBM with high *CD276* expression may be resistant to lomustine and nitrosourea. Through a comprehensive evaluation of *CD276*, we have also shown that *CD276* expression is closely correlated with tumor immune regulation, immune cell infiltration and immune checkpoint responses, which has provided important insights into the future optimization of cancer immunotherapy utilizing the *CD276* pathway.

## Supplementary Information

**Additional file 1: Supplementary Fig. S1.** Pan-cancer paired CD276 expression. (A) The expression of CD276 in cancer and corresponding normal tissues. (B-P) TCGA database was applied to investigate the expression of CD276 in paired cancer tissues and corresponding adjacent normal tissues. The paired tissues with significant differences and statistical significance were listed in the figure. *p < 0.05; **p < 0.01 and ***p < 0.001. **Supplementary Fig. S2.** Analysis of the relationship between CD276 expression and clinicopathological parameters in cancers. (A-T) The clinical relevance of CD276 in different cancer stages was analyzed using UALCAN database. **Supplementary Fig. S3.** Analysis of the relevance between CD276 expression and clinicopathological parameters in different cancers. (A-F) The clinical correlation of CD276 in different cancer stages was analyzed using UALCAN database, and the expression of CD276 in different cancer stages shown in the Fig. was not statistically significant. **Supplementary Fig. S4.** Analysis of DNA methylation in pan-cancer. (A-I) UALCAN database was used to explore promoter methylation levels of CD276 in different cancers, and the cancers selected in the Fig. showed statistical differences between tumors and normal tissues. **Supplementary Fig. S5.** CD276 is highly expressed in many cancers and normal tissues. Immunohistochemical results of HPA dataset showed that protein expression of CD276 was high in various cancers, just as shown in Fig. A-E, and protein expression of CD276 was also high in various normal tissues, just as shown in Fig. F-I. **Supplementary Fig. S6.** After constructing PPI network, we obtained a series of proteins closely related to CD276. DAVID database was used to conduct GO and KEGG enrichment analysis on CD276 and its related genes, so as to explore the biological functions associated with CD276. **Supplementary Fig. S7.** Merged enrichment plots for CD276 on the basis of GSEA in pan-cancer. Fig. A-H shows the signaling pathways associated with CD276 by GO, KEGG and Reactome analysis, including those involved in neutrophil degranulation and signaling by interleukins. **Supplementary Fig. S8.** The relationship between CD276 and immunoregulation-related genes. The relevance between the expression of CD276 and immune-activating genes, immunosuppressive genes, chemokine receptors and chemokines were analyzed using TISDB database. **Supplementary Fig. S9.** The expression and prognostic value of CD276 in GBM. A-B. The mRNA expression of CD276 in GBM using TCGA, E-MATB and GEO databases; C. The protein expression of CD276 in GBM. D-E. The prognostic value of CD276 in GBM using CGGA database.

## Data Availability

All data utilized in this study are included in the article and all data are available on reasonable request from the corresponding author. Direct web links of datasets about; Human Cell Landscape: http://bis.zju.edu.cn/HCL; PanglaoDB: https://panglaodb.se/index.html; Tabula Muris:https://tabula-muris.ds.czbiohub.org/; CancerSEA: http://biocc.hrbmu.edu.cn/CancerSEA/home.jsp; TIGER: http://tiger.canceromics.org/; TCGA: https://www.cancer.gov/; GTEx: https://commonfund.nih.gov/GTEx/; cBioPortal: https://www.cbioportal.org/; HPA: https://www.proteinatlas.org/; GPS-Prot: http://gpsprot.org/; GeneMANIA: http://genemania.org/; STRING: https://string-db.org/; DAVID: https://david.ncifcrf.gov/; UALCAN; http://ualcan.path.uab.edu/; DiseaseMeth version 2.0: http://bio-bigdata.hrbmu.edu.cn/diseasemeth/; MEXPRESS: https://mexpress.be; MethSurv: https://biit.cs.ut.ee/methsurv/; Kaplan-Meier Plotter: http://www.kmplot.com/analysis/; TIMER2.0: http://timer.cistrome.org/; TISDB: http://cis.hku.hk/TISIDB/index.php; GDC: https://gdc.cancer.gov/; LinkedOmics: http://www.linkedomics.org/login.php; ROC plotter: http://rocplot.org/; PubChem: https://pubchem.ncbi.nlm.nih.gov/; Sangerbox 3.0: http://sangerbox.com/home.html.

## Abbreviations

ACC: Acute Myeloid Leukemia
BLCA: Bladder Urothelial Carcinoma
BRCA: Breast invasive carcinoma
CESC: Cervical squamous cell carcinoma and endocervical adenocarcinoma
CHOL: Cholangiocarcinoma
COAD: Colon adenocarcinoma
ESCA: Esophageal carcinoma
GBM: Glioblastoma multiforme
HNSC: Head and Neck squamous cell carcinoma
KICH: Kidney Chromophobe
KIRC: Kidney renal clear cell carcinoma
KIRP: Kidney renal papillary cell carcinoma
LAML: Acute Myeloid Leukemia
LIHC: Liver hepatocellular carcinoma
LGG: Lower Grade Glioma
LUAD: Lung adenocarcinoma
LUSC: Lung squamous cell carcinoma
MESO: Mesothelioma
OV: Ovarian serous cystadenocarcinoma
PAAD: Pancreatic adenocarcinoma
PCPG: Pheochromocytoma and Paraganglioma
PRAD: Prostate adenocarcinoma
READ: Rectum adenocarcinoma
SARC: Sarcoma
SKCM: Skin Cutaneous Melanoma
STAD: Stomach adenocarcinoma
TGCT: Testicular Germ Cell Tumor
THYM: Thymoma
THCA: Thyroid carcinoma
UCS: Uterine Carcinosarcoma
UCEC: Uterine Corpus Endometrial Carcinoma
UVM: Uveal Melanoma
AML: Acute Myeloid Leukemia
RYK: receptor protein tyrosine kinases
CLEC5A: Spleen tyrosine kinase (syk)-coupled C-type lectins 5A
RNF4: Recombinant Human Ring Finger Protein 4
LGALS8: The galectin-8 gene
LGALS9: The galectin-9 gene
PVR: poliovirus receptor
ENTPD1: ectonucleoside triphosphate diphosphohydrolase 1
NT5E: ecto-5'-nucleotidase
TGFBR1: transforming growth factor beta (TGF-beta) receptors 1
TGFB1: transforming growth factor beta 1
NECTIN2: Poliovirus Receptor-Related Protein 2
IL10RB: Interleukin 10 Receptor Beta
CSF1R: Colony stimulating factor-1 receptor
CCR1: chemokine (C-C motif) receptor 1
CCL7: Chemokine (C-C motif) ligand 7
CXCL16: CXC chemokine ligand 16
CXCL8: CXC chemokine ligand 8
