# Supplementary material for: Multi-omics analyses of CD276 in pan-cancer reveals its clinical prognostic value in glioblastoma and other major cancer types
Source: BMC Cancer. 2023 Jan 30;23:102. doi: 10.1186/s12885-023-10575-1 (PMC9885708; doi:10.1186/s12885-023-10575-1)
Supplement: Supplementary file 1 — Additional file 1: Supplementary Fig. S1. Pan-cancer paired CD276 expression. (A) The expression of CD276 in cancer and corresponding normal tissues. (B-P) TCGA database was applied to investigate the expression of CD276 in paired cancer tissues and corresponding adjacent normal tissues. The paired tissues with significant differences and statistical significance were listed in the figure. *p < 0.05; **p < 0.01 and ***p < 0.001. Supplementary Fig. S2. Analysis of the relationship between CD276 expression and clinicopathological parameters in cancers. (A-T) The clinical relevance of CD276 in different cancer stages was analyzed using UALCAN database. Supplementary Fig. S3. Analysis of the relevance between CD276 expression and clinicopathological parameters in different cancers. (A-F) The clinical correlation of CD276 in different cancer stages was analyzed using UALCAN database, and the expression of CD276 in different cancer stages shown in the Fig. was not statistically significant. Supplementary Fig. S4. Analysis of DNA methylation in pan-cancer. (A-I) UALCAN database was used to explore promoter methylation levels of CD276 in different cancers, and the cancers selected in the Fig. showed statistical differences between tumors and normal tissues. Supplementary Fig. S5. CD276 is highly expressed in many cancers and normal tissues. Immunohistochemical results of HPA dataset showed that protein expression of CD276 was high in various cancers, just as shown in Fig. A-E, and protein expression of CD276 was also high in various normal tissues, just as shown in Fig. F-I. Supplementary Fig. S6. After constructing PPI network, we obtained a series of proteins closely related to CD276. DAVID database was used to conduct GO and KEGG enrichment analysis on CD276 and its related genes, so as to explore the biological functions associated with CD276. Supplementary Fig. S7. Merged enrichment plots for CD276 on the basis of GSEA in pan-cancer. Fig. A-H shows the signaling pathwa [file 12885_2023_10575_MOESM1_ESM.docx]

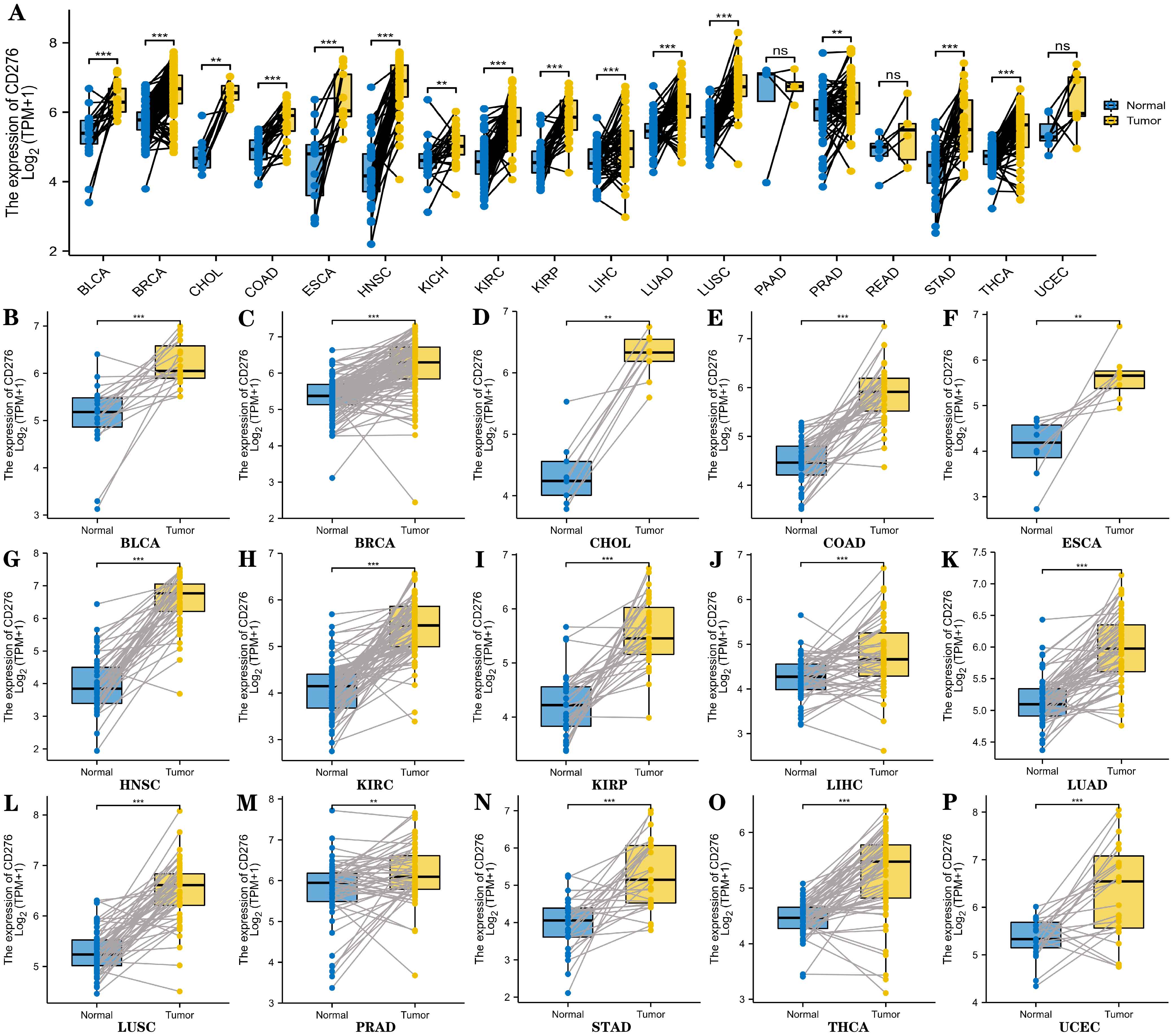
**Fig. supplementary Fig. S1** Pan-cancer paired CD276 expression. (A) The expression of CD276 in cancer and corresponding normal tissues. (B-P) TCGA database was applied to investigate the expression of CD276 in paired cancer tissues and corresponding adjacent normal tissues. The paired tissues with significant differences and statistical significance were listed in the figure. *p < 0.05; **p < 0.01 and ***p < 0.001.


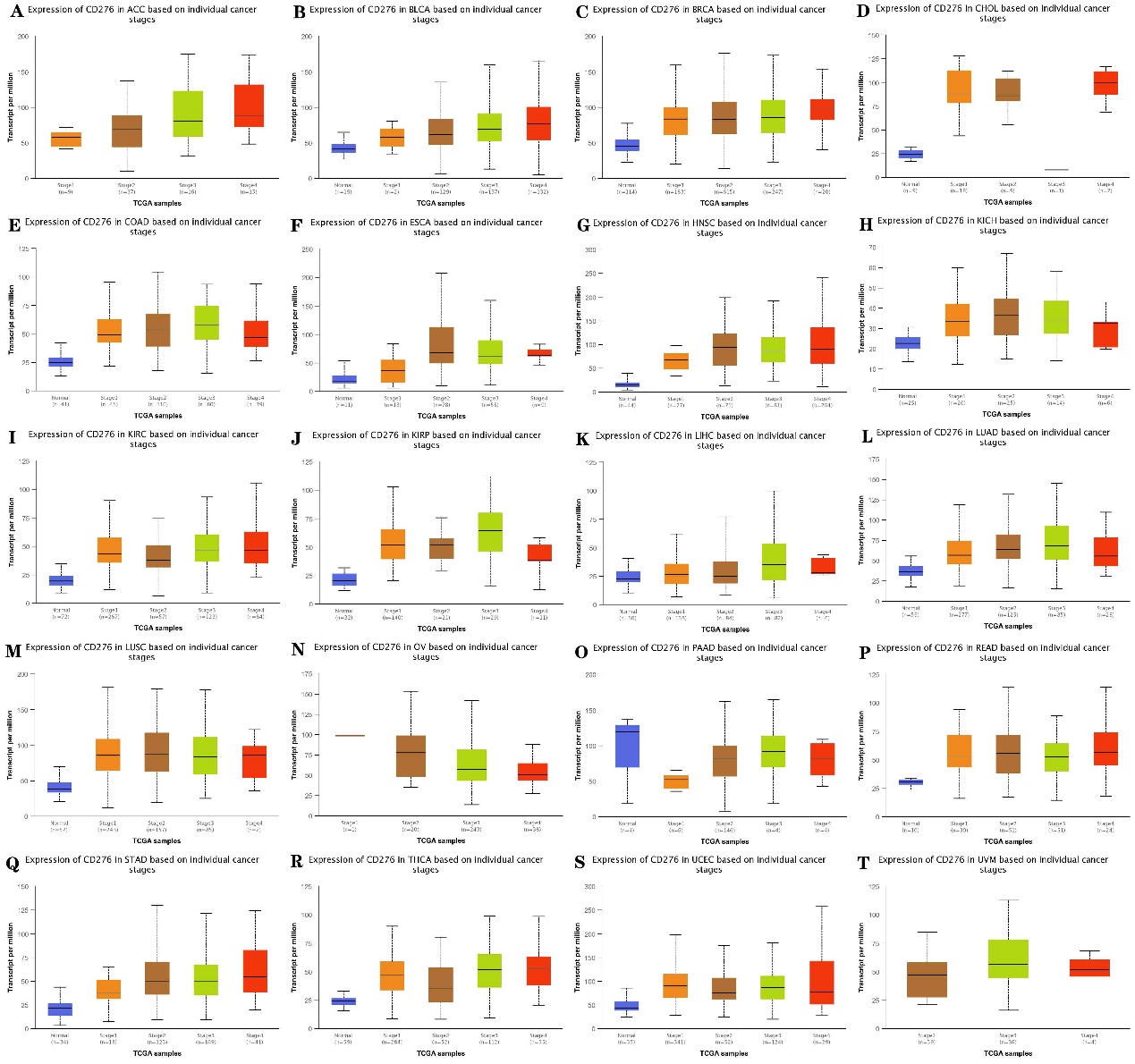


**Fig. supplementary Fig. S2** Analysis of the relationship between CD276 expression and clinicopathological parameters in cancers. (A-T) The clinical relevance of CD276 in different cancer stages was analyzed using UALCAN database.


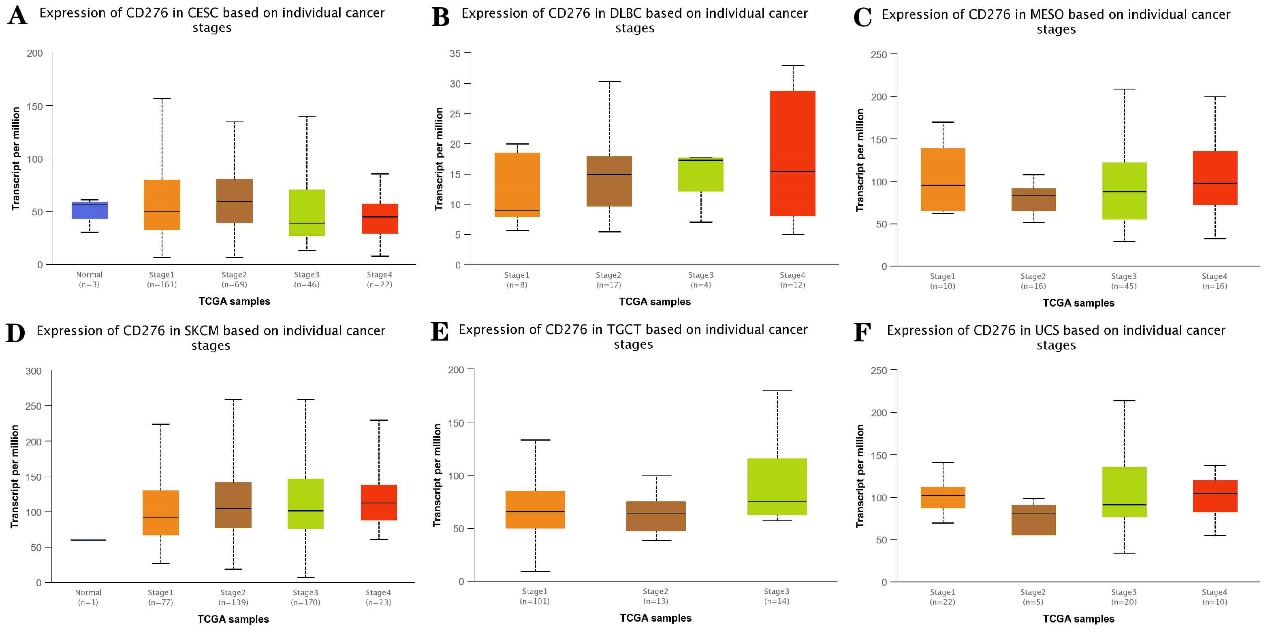


**Fig. supplementary Fig. S3** Analysis of the relevance between CD276 expression and clinicopathological parameters in different cancers. (A-F) The clinical correlation of CD276 in different cancer stages was analyzed using UALCAN database, and the expression of CD276 in different cancer stages shown in the Fig. was not statistically significant.


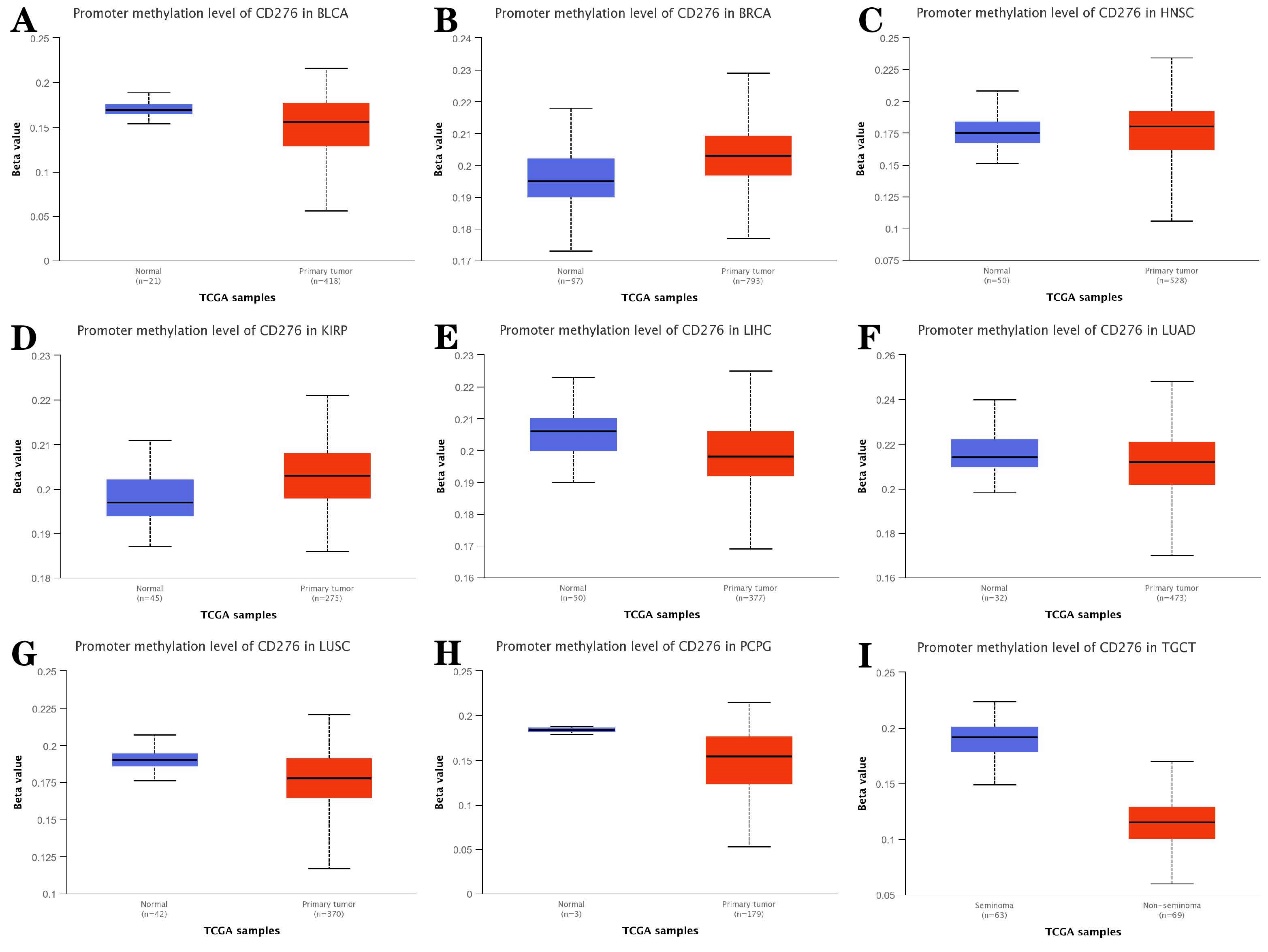


**Fig. supplementary Fig. S4** Analysis of DNA methylation in pan-cancer. (A-I) UALCAN database was used to explore promoter methylation levels of CD276 in different cancers, and the cancers selected in the Fig. showed statistical differences between tumors and normal tissues.


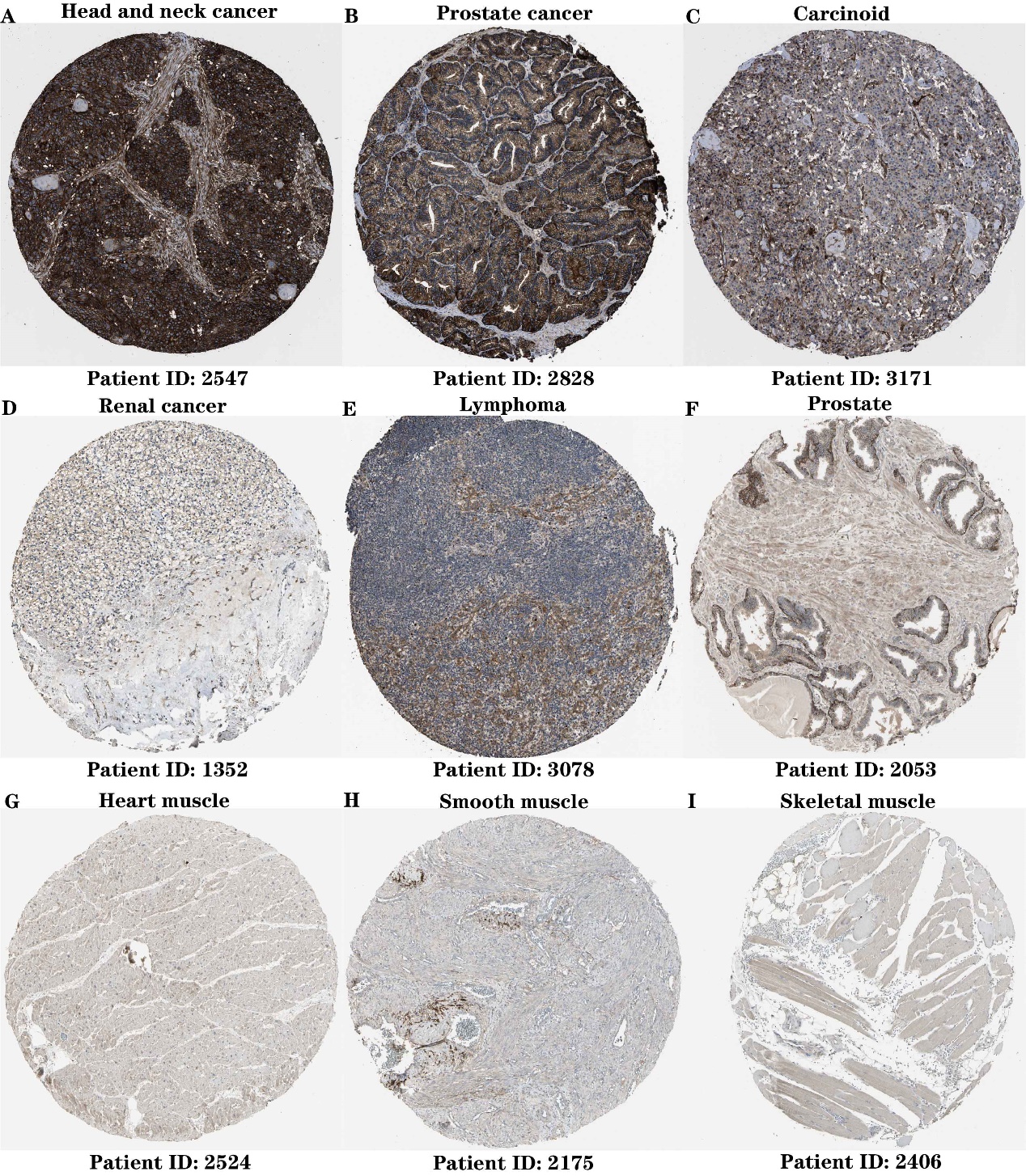


**Fig. supplementary Fig. S5** CD276 is highly expressed in many cancers and normal tissues. Immunohistochemical results of HPA dataset showed that protein expression of CD276 was high in various cancers, just as shown in Fig. A-E, and protein expression of CD276 was also high in various normal tissues, just as shown in Fig. F-I.


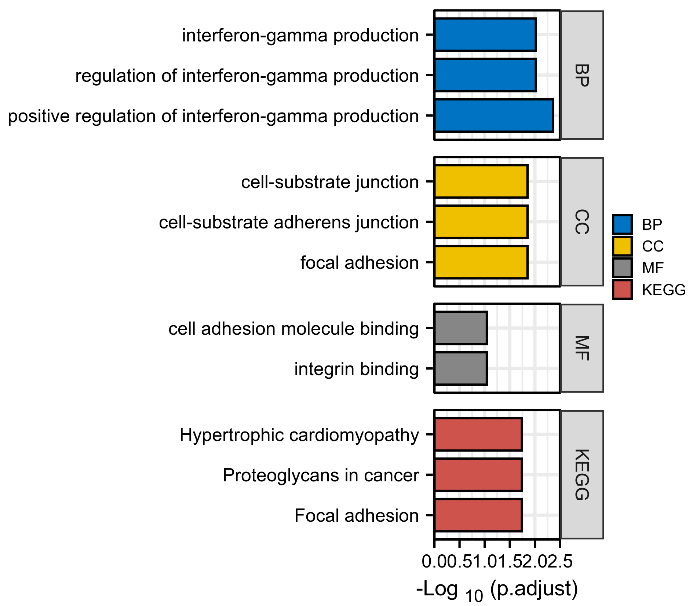


**Fig. supplementary Fig. S6** After constructing PPI network, we obtained a series of proteins closely related to CD276. DAVID database was used to conduct GO and KEGG enrichment analysis on CD276 and its related genes, so as to explore the biological functions associated with CD276


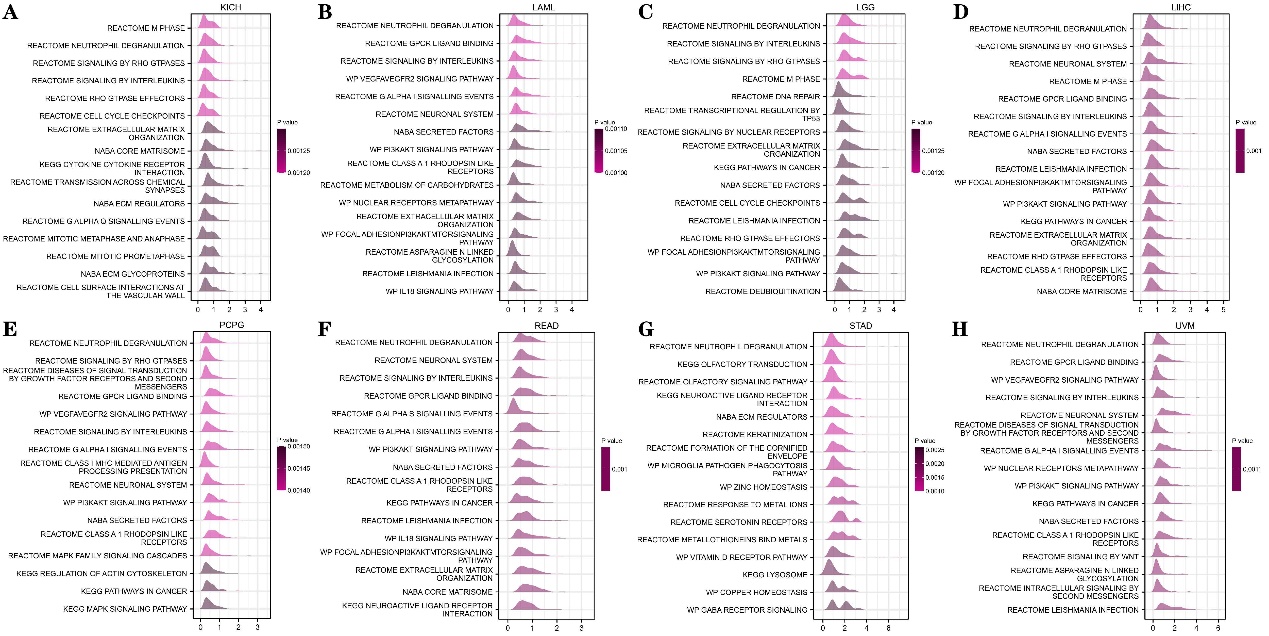


**Fig. supplementary Fig. S7** Merged enrichment plots for CD276 on the basis of GSEA in pan-cancer. Fig. A-H shows the signaling pathways associated with CD276 by GO, KEGG and Reactome analysis, including those involved in neutrophil degranulation and signaling by interleukins.


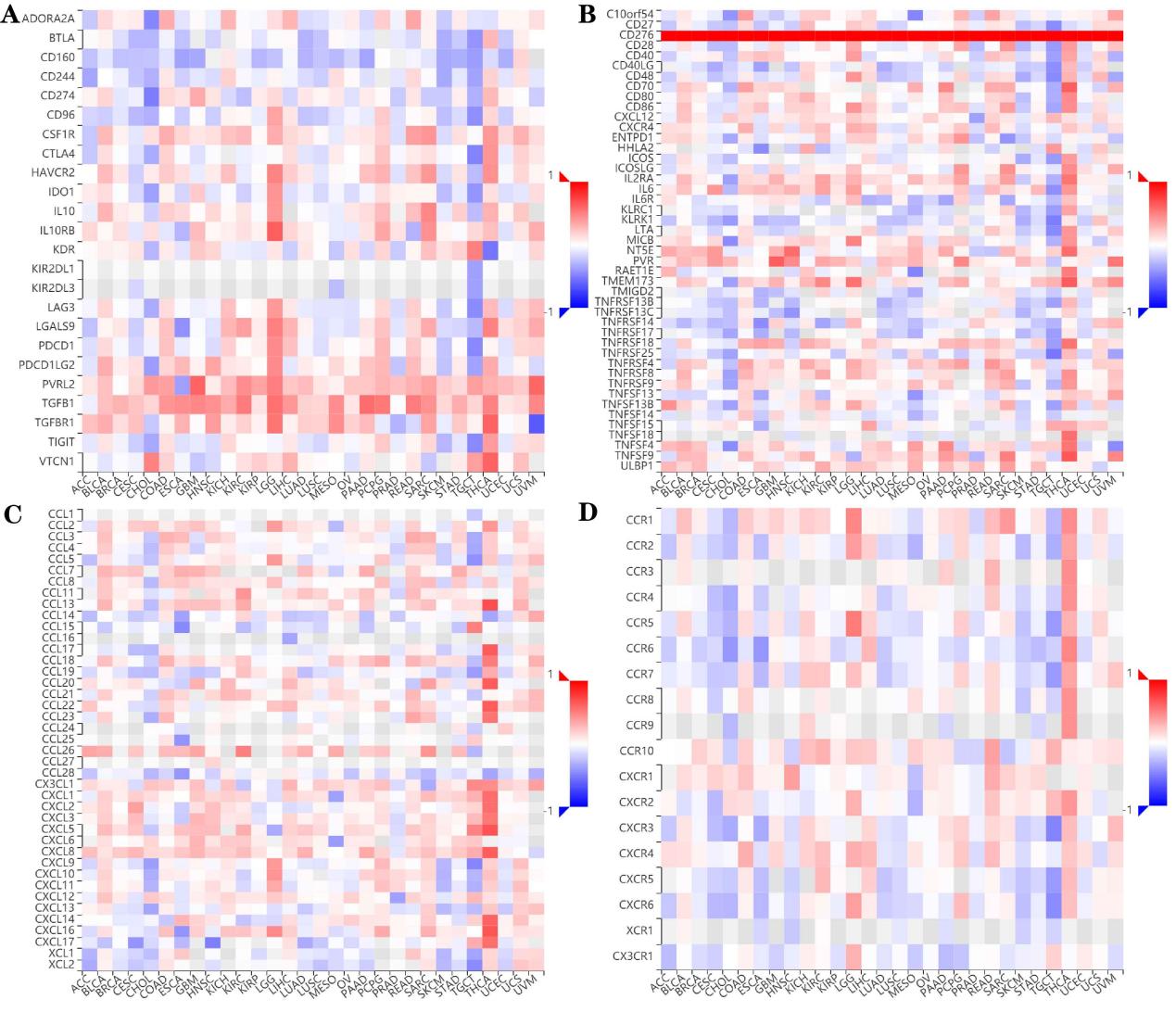


**Fig. supplementary Fig. S8** The relationship between CD276 and immunoregulation-related genes. The relevance between the expression of CD276 and immune-activating genes, immunosuppressive genes, chemokine receptors and chemokines were analyzed using TISDB database.


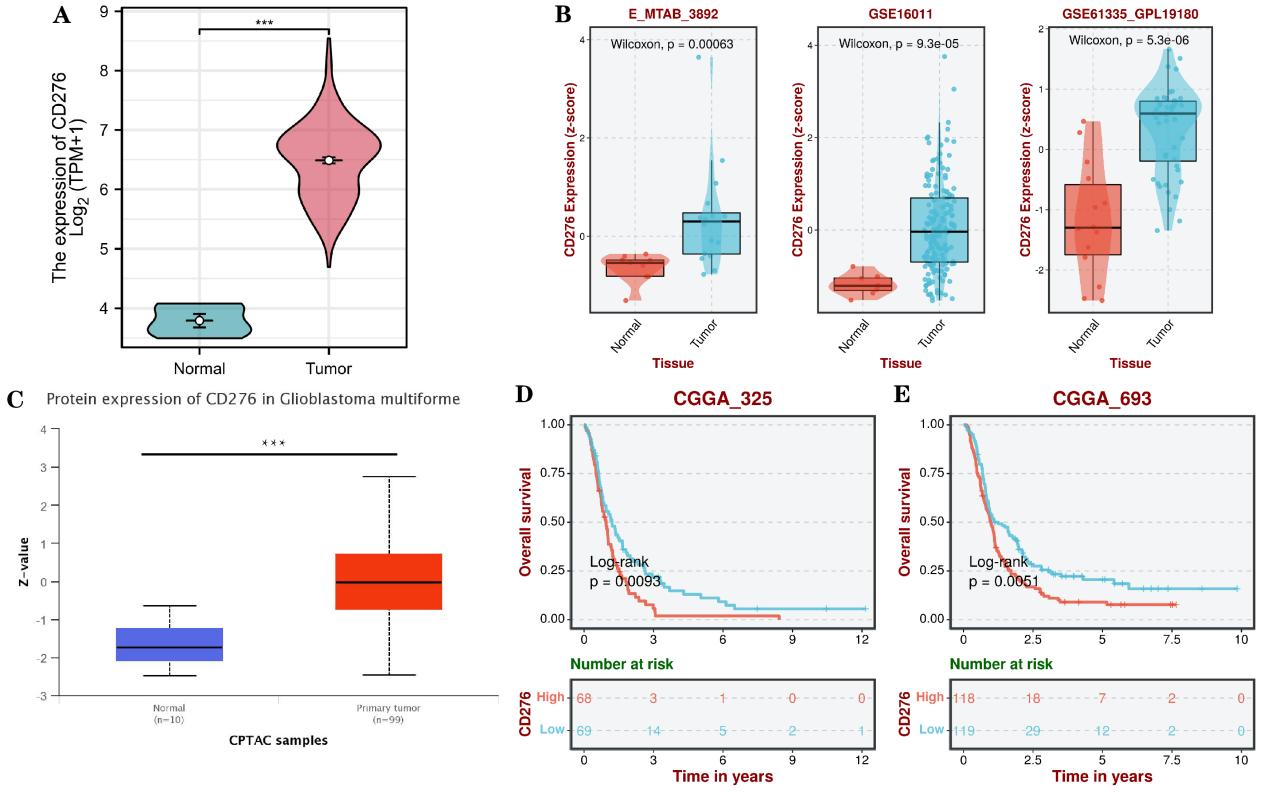


**Fig. supplementary Fig. S9** The expression and prognostic value of CD276 in GBM. A-B. The mRNA expression of CD276 in GBM using TCGA, E-MATB and GEO databases; C. The protein expression of CD276 in GBM. D-E. The prognostic value of CD276 in GBM using CGGA database.
